# Metabolic reprogramming of poly(morpho)nuclear giant cells determines glioblastoma recovery from doxorubicin-induced stress

**DOI:** 10.1186/s12967-024-05541-9

**Published:** 2024-08-12

**Authors:** Maciej Pudełek, Damian Ryszawy, Katarzyna Piwowarczyk, Sławomir Lasota, Zbigniew Madeja, Sylwia Kędracka-Krok, Jarosław Czyż

**Affiliations:** 1 Department of Cell Biology, Faculty of Biochemistry, Biophysics and Biotechnology, Jagiellonian University, Gronostajowa 7, Krakow, 30-387 Poland; 2 Doctoral School of Exact and Natural Sciences, Jagiellonian University, Krakow, Poland; 3 Department of Physical Biochemistry, Faculty of Biochemistry, Biophysics and Biotechnology, Jagiellonian University, Kraków, Poland

**Keywords:** Glioblastoma multiforme, Doxorubicin, Polymorphonuclear giant cells, Metabolism, Drug-resistance, Microevolution

## Abstract

**Background:**

Multi-drug resistance of poly(morpho)nuclear giant cells (PGCs) determines their cytoprotective and generative potential in cancer ecosystems. However, mechanisms underlying the involvement of PGCs in glioblastoma multiforme (GBM) adaptation to chemotherapeutic regimes remain largely obscure. In particular, metabolic reprogramming of PGCs has not yet been considered in terms of GBM recovery from doxorubicin (DOX)-induced stress.

**Methods:**

Long-term proteomic and metabolic cell profiling was applied to trace the phenotypic dynamics of GBM populations subjected to pulse DOX treatment in vitro, with a particular focus on PGC formation and its metabolic background. The links between metabolic reprogramming, drug resistance and drug retention capacity of PGCs were assessed, along with their significance for GBM recovery from DOX-induced stress.

**Results:**

Pulse DOX treatment triggered the transient formation of PGCs, followed by the appearance of small expanding cell (SEC) clusters. Development of PGCs was accompanied by the mobilization of their metabolic proteome, transient induction of oxidative phosphorylation (OXPHOS), and differential intracellular accumulation of NADH, NADPH, and ATP. The metabolic background of PGC formation was confirmed by the attenuation of GBM recovery from DOX-induced stress following the chemical inhibition of GSK-3β, OXPHOS, and the pentose phosphate pathway. Concurrently, the mobilization of reactive oxygen species (ROS) scavenging systems and fine-tuning of NADPH-dependent ROS production systems in PGCs was observed. These processes were accompanied by perinuclear mobilization of ABCB1 and ABCG2 transporters and DOX retention in the perinuclear PGC compartments.

**Conclusions:**

These data demonstrate the cooperative pattern of GBM recovery from DOX-induced stress and the crucial role of metabolic reprogramming of PGCs in this process. Metabolic reprogramming enhances the efficiency of self-defense systems and increases the DOX retention capacity of PGCs, potentially reducing DOX bioavailability in the proximity of SECs. Consequently, the modulation of PGC metabolism is highlighted as a potential target for intervention in glioblastoma treatment.

**Supplementary Information:**

The online version contains supplementary material available at 10.1186/s12967-024-05541-9.

## Background

Glioblastoma multiforme (GBM; IV-grade brain tumor/glioma; WHO) is the most aggressive subtype of glioma [1, 2]. Basic regimens applied for GBM treatment include radiotherapy, surgical tumor resection and chemotherapy [3]. However, brain anatomy [4–6] and the presence of blood-brain barriers [7, 8] interfere with their efficiency. Moreover, chemotherapy-induced microevolution of tumor cells towards the drug-resistant phenotype results in the recurrences of GBM tumors after the cessation of standard chemotherapy [9–11]. Consequently, the average survival time of GBM patients has only slightly increased over the last 30 years. It ranges from 12 to 14 months [12, 13] and less than 5% of GBM patients survive 5 years after diagnosis [14, 15]. Despite the development of GBM treatment strategies in the recent decades, these figures remain a constant challenge for the contemporary neuro-oncology [16].

High hopes to overcome the problem of GBM microevolution and recurrence after chemotherapy have been kindled by the introduction of doxorubicin (DOX) into routine GBM treatment. Due to its cytostatic, pro-apoptotic and anti-invasive activity, DOX has been successfully introduced in the chemotherapy of leukemia, breast, and lung cancer [17]. Therapeutic advantage of this anthracycline antibiotic relies on the spectrum of activities, which include the deterioration of genome integrity, interference with the calcium homeostasis and the induction of oxidative stress [18–21]. However, promising results of the attempts to introduce DOX into GBM therapy have been confronted with an array of reports on the limitations and side-effects of this strategy [21, 22]. Apart from DOX cardiotoxicity and limited ability to penetrate blood-brain barriers (recently overcome by the application of carriers [23–29]), glioma cell adaptation to DOX-induced stress limits the efficiency of DOX in glioma treatment [9]. On the other hand, the mechanisms that govern the onset of this process and the cooperation of discrete cell lineages during DOX-induced GBM adaptation have not been considered. This limits our knowledge on the potential pitfalls of DOX application in neuro-oncology.

At the population level, GBM responses to DOX-induced stress are governed by the balance between the negative “Darwinian” and positive “Lamarckian” selection [30–35]. Chemotherapy eliminates stress-sensitive cells, which are gradually overgrown by stress-resistant cells. They are usually characterized by mobilized ABC transporters, drug-inactivating enzymes and intracellular repair systems, and by enhanced metabolic plasticity [18, 36, 37]. The combined epigenetic and genetic events, which enhance the adaptive capabilities of cancer cells, include the DNA/histone methylation/acetylation, mutations, chromosomal rearrangements and whole-genome changes. In particular, polyploidy secures a DNA pool for transcriptional and translational management of self-defense systems that sustain the detoxifying abilities of poly(morpho)nuclear giant cells (PGCs) [38–40]. The role of PGCs in cancer recovery from chemotherapeutic stress is prevalently ascribed to de novo formation of multipotent, diploid progeny (neosis; [41]). However, cancer adaptation to chemotherapeutic stress can also be considered as a role-playing game, where the dormant PGCs act as “chaperon” cells to serve and protect residual (sub)population(s) of expansive cells. PGCs have been observed in histological glioblastoma biopsies and their role in glioma progression acknowledged [42, 43]. However, the involvement of PGCs in the adaptation of glioma cell populations to DOX-induced stress remains unaddressed.

Similarly, the significance of metabolic reprogramming for the protective potential of PGCs has not yet been analyzed. The Warburg effect (induction of aerobic glycolysis) is often linked to the drug resistance of invasive cancer cell lineages [44]. In turn, numerous data describe oxidative phosphorylation (OXPHOS) as the principal energy source in drug-resistant cancer cells [45–50]. It remains to be elucidated how metabolic plasticity and reprogramming [51] contribute to the formation, self-protective activities, and “chaperone” functions of PGCs. Preliminary data have shown that a pulse DOX treatment triggers a two-phasic adaptation process in the populations of the model GBM T98G cells. In its first phase, the “selective elimination” of drug-sensitive cells precedes the generation of dormant PGCs from their drug-resistant counterparts. PGCs further contribute to the microevolution of small expanding cells (SECs) during the second (relapse) phase of this process. This experimental model was adopted to trace the PGC-dependent scenario(s) of GBM recovery from DOX-induced stress. In particular, (i) processes underlying DOX-induced PGC formation and phenotypic diversification of GBM populations were addressed along with (ii) their metabolic background. Next, (iii) determinants of remarkable PGC resistance to long-term DOX retention were analyzed together with (iv) their significance for the recovery of GBM populations from DOX-induced stress.

## Materials and methods

### Cell culture

Human glioma T98G (ATCC, CRL-1690), U87-MG (ATCC, HTB-14), Ln18 (ATCC, CRL-2610) and Ln229 (ATCC, CRL-2611) cells were cultured in the standard conditions (37^o^C, 5% CO_2_) in the high glucose (4500 g/L) DMEM medium (Sigma, No. D6429), supplemented with 10% heat-inactivated fetal bovine serum (FBS; Gibco, No. A3840402), 0.2% Plasmocin^®^ prophylactic (Invivogen; No. ant-mpp) and 1% Antibiotic-Antimycotic Solution (Merck, No. A5955; 100 units penicillin, 0.1 mg streptomycin, 0.25 µg amphotericin B) as described previously [52]. Cell cultures were routinely tested for mycoplasma using a MycoStrip™ Mycoplasma detection kit (Invivogen; No. rep-mys). The cells were harvested with Ca^2+^/Mg^2+^-free DPBS/0.5 mM UlraPure™ EDTA solution (Invitrogen, No. 15575020; Invitrogen, No. 14190144), counted in Z2 particle counter (Beckman Coulter) and seeded into multi-well tissue culture plates (Falcon^®^). Unless stated otherwise, the cells were exposed to pulse doxorubicin treatment for 48 h (DOX, 0.01-1 µM; Sigma, No. D1515; prepared from 10 mM DOX stock solution in sterile DMSO; Sigma, No. D8418) and rinsed with the fresh medium to remove unattached (dead) cells prior to the experiment. Analyses of long-term DOX effects were performed at the indicated time-points (72 h-50 days) after DOX removal. Cells cultured in the presence of 0.1% DMSO were used as control samples. Where indicated, the cells were treated with 3 µM Cis-Platin (Cis-Pt, Sigma; No. P4394; from 10 mM stock in DMSO), 10 µM paclitaxel (PTX; Sigma; No. T7402; from 10 mM stock in DMSO), 250 µM carmustine (Sigma; No. C0400; from 250 mM stock in DMSO), 5 µM 5-fluorouracil (5-FU, Sigma; No. F6627; from 5 mM stock in DMSO), 10–50 µM TWS119 (GSK-3β inhibitor, Sigma; No. SML1271; from 25 mM stocks in DMSO), 5 µM Oligomycin A (OliA, ATP synthase inhibitor, Sigma; No. 7535, from 5 mM stock in DMSO), 250 µM Etomoxir (ETX, β-oxidation inhibitor; Sigma; No. 236020, 2023 from 250 mM stock in DMSO), 5 µM 6-aminonicotinamide (6-ANA, pentose-phosphate pathway inhibitor; from 100 mM stock DMSO), 250–1000 µM sodium ascorbate (Asc, Sigma; No. A4034, from 500 mM stock in PBS), 1–5 mM N-acetyl-L-cysteine (NAC, Sigma; No. A9165; from 5 M stock in PBS), 1–25 mM Taurine (Tau, Sigma; No. T8691; from 5 mM stock in PBS) and 10-1000 µM hydrogen peroxide (H_2_O_2_, POCH; No. 885193111; from freshly prepared 100 mM stock in culture medium). No state or ethical approval was required for this study. No patients consent was required for this study.

### Morphometry and fluorimetry

For morphometric analyses, adherent 3.7% formaldehyde-fixed cells or their Hoechst 33,258-stained nuclei were visualized with the IMC optics (Hoffman contrast) or epifluorescence, respectively. Then, their contours were manually drawn and cell/nuclear surface areas, elongation (aspect ratio, i.e., the ratio of the major to minor axis of an elliptical fit depicting rear-front polarity) and circularity (i.e., the ratio of area and perimeter) were quantified with ImageJ software. The contours of at least 25 cells was analyzed for each condition. Fraction of mesenchymal cells was estimated according to cell elongation/circularity values (> 2.5 and < 0.5, respectively). Cell volumes were estimated based on the radiuses of cell projection areas in suspension, quantified using fluorescence microscopy (Leica) and ImageJ software. For fluorimetric analyses, the images of 4–6 randomly selected culture regions were collected for each condition with the same excitation/exposure settings (excitation/camera gain/time of exposition). Fluorescence intensities were estimated with ImageJ software and normalized against cell number/volume [52].

### Cell migration and transmigration

Analyses of cell movement were performed in 12-well plates (Falcon^®^). Cells were seeded at the density of 2 × 10^4^ cells/well, subjected to the protocol of pulse DOX treatment/regeneration and visualized with time-lapse videomicroscopy at the indicated time-points. Their movement was recorded for 8 h with 5 min. time interval using Leica DMI6000B system equipped with the integrated modulation contrast (IMC; Hoffman contrast), CO_2_ (5%) and temperature (37^o^C) monitoring system. The sequences of images were analyzed with Hiro v.1.0.0.4 software (written by W. Czapla) by manual cell trajectory tracking, followed by the calculation of cell motility parameters (speed of movement [µm/min] and displacement [µm]) [53]. Time-lapse images were further used for the classification of cell morphology. Invasive potential of the cells was examined with the transmigration assay (Transwell™ microporous (8 μm) membranes; Corning^®^). Cells were seeded onto the upper layers of membranes at the density of 2 × 10^4^ cells/insert and allowed to transmigrate in the presence/absence of 1µM DOX for 24–96 h. Then, transmigrated cells were harvested with TrypLE and counted with Z2 particle counter. Transmigration index (TMI) was calculated as the % of cells that managed to penetrate micropores within the transmigration time (24–96 h) [52, 55].

### Calcein efflux assay

The cells in 12-well culture plates (Corning^®^Costar^®^) were loaded with 1 µg/ml Calcein-AM (Invitrogen, No. C3099) for 30 min. Then, the medium was replaced for FluoroBrite^®^ DMEM (Gibco^®^; No. A1896701; supplemented with 10% FBS and 1% GlutaMAX; Gibco^®^; No. A1286001). Initial (t_0_) intracellular calcein fluorescence intensity and its changes over the time (which illustrate calcein efflux) were monitored with Leica DMI6000B fluorescence system (see above), using Alexa488 filter set and time-lapse imaging module (time step = 30 min.; total acquisition time = 120 min.). Images were processed in ImageJ software [54].

### Immunofluorescence and immunoblotting

For immunofluorescence studies, the cells were seeded into 12-well plates on UVC-sterilized coverslips at the density of 2 × 10^4^ cells/well, cultured for 24 h and subjected to the protocol of pulse DOX treatment/regeneration. Then, they were fixed with 3.7% formaldehyde followed by 0.1% Triton X-100 permeabilisation [52]. Non-specific binding sites were blocked with 2% BSA (Invitrogen, No. 37525; 30 min. in 37^o^C). Specimens were incubated for 45 min. in the presence of the following primary antibodies (in 2% BSA/0.01% Tween): polyclonal goat anti-SNAI1 (N-terminal, Sigma; No. SAB2501370; 1:300), polyclonal rabbit anti-Cx43 (Sigma; No. C6219; 1:500), monoclonal IgG mouse anti-α-tubulin (Sigma; No. T6199; 1:300), polyclonal rabbit anti vimentin (GeneTex; No. GTX100619; 1:200) and polyclonal rabbit anti phospho-Snail-1-PSer246 (Sigma; No. SAB4504319; 1:200) and polyclonal rabbit anti-Ki67 (Sigma; No. SAB5700770; 1:300). Immunolocalization of stress resistance-related proteins was performed with polyclonal rabbit anti-ABCB1 (Sigma; No. HPA002199; 1:250) and monoclonal rabbit anti-ABCG2 (Sigma; No. ZRB1217; 1:100), polyclonal rabbit anti-MnSOD (Sigma; No. HPA001814, 1:300), polyclonal rabbit anti-glutathione synthetase (GSS; ABclonal; No. A14535, 1:200), monoclonal rabbit anti-glycogen synthase kinase-3 beta (GSK-3β) (ABclonal; No. A11731, 1:200) and polyclonal rabbit anti-MTCO2 antibody (Invitrogen; No. MA5-12017; 1:100). After washing with 2% BSA, the cocktails of the following secondary antibodies (different combinations in 2% BSA/0.01% Tween; 1:500) were applied for 45 min.: AlexaFluor488-conjugated chicken anti-goat (Invitrogen; No. A21467), AlexaFluor488-conjugated chicken anti-rabbit IgG (Invitrogen; No. A21441), AlexaFluor488-conjugated donkey anti-mouse IgG (Invitrogen; No. A21202), AlexaFluor647-conjugated chicken anti-rabbit IgG (Invitrogen; No. A21443), AlexaFluor546-conjugated donkey anti-mouse IgG (Invitrogen; A10036), AlexaFluor546-conjugated phalloidin (Invitrogen, No. A22283; for F-actin visualization; 1:80) and Hoechst 33,258 (Sigma; for DNA staining; 1–2 µg/ml). Afterwards, specimens were mounted in Moviol 4–88 mounting medium or ProLong™ Gold Antifade Mountant (Invitrogen; No. P10144). Images were acquired with Leica DMI6000B fluorescence microscope equipped with DFC360FX CCD camera and total internal reflection fluorescence (TIRF) module or Leica Stellaris 5 confocal microscope.

For the estimation of intracellular Cx43 levels, the cells were harvested with the cold (~ 4^o^C) Ca^2+^/Mg^2+^-free PBS/EDTA, centrifuged and dissolved in protease inhibitor cocktail/lysis buffer, followed by their freeze-thawing/sonication. Bradford assay was used for the determination of total protein content in the samples. Protein samples (20 µg) were subjected to SDS-PAGE electrophoresis on 12% polyacrylamide gel (Laemmli protocol), followed by their transfer to PVDF membranes (Immun-Blot^®^ PVDF Membrane, #1620177; Bio-Rad) and blocking of unspecific staining with the skimmed milk/TBST solution. For protein immunodetection, monoclonal polyclonal rabbit anti-Cx43 IgG (No. C6219; 1:3000), mouse anti-α-tubulin IgG (No. T9026; 1:1000), HRP-conjugated goat anti-rabbit IgG (Thermo Fisher Scientific; No. 31466) and HRP-conjugated goat anti-mouse IgG (Thermo Fisher Scientific; No. 31430) were used (all from Sigma). Signal detection (HRP substrate; Merck, Luminata Crescendo; No. WBLUR0500) was performed with the MicroChemii system (SNR Bio-Imaging System; [52]).

### Viability assay

For the estimation of cell viability and proliferation, the cells were seeded into 12–well cell culture plates (Corning^®^Costar^®^) at the density of 2 × 10^4^ cells/well and subjected to the protocol of pulse DOX treatment/regeneration. Then, the cells were dissociated, resuspended in original medium and subjected to Trypan blue (Sigma; No. T8154) inclusion assay (with Bürker haemocytometer; Marienfeld) at the indicated time-points. EC50 values (after 72 h of incubation with DOX) were calculated with Quest Graph™ EC50 Calculator [AAT Bioquest, Inc.].

### Calcein microinjection

Cells were seeded into µ-Dish 35 mm, high (Ibidi; No. 81156) at the density of 2 × 10^4^ cells/well for 24 h and subjected to pulse DOX treatment (1 µM; 48 h). 14 days afterwards, the standard medium was changed to FluoroBrite™ DMEM (supplemented as described above) followed by selective microinjection of calcein (Sigma; No. C0875; 2023, 0.1 mg/ml in PBS, centrifuged and sterile filtered with 0.22 μm syringe filter) into poly(morpho)nuclear giant cells (PGCs; injection pressure = 140 hPa; injection time 2–6 s; compensation pressure 15 hPa; InjectMan^®^ 4 and FemtoJet^®^ 4; Eppendorf). Time-lapse monitoring of the calcein flux from PGCs to SECs was performed for 30 min (time step: 3 min; 5% CO_2_; 37^o^C; Alexa488 filter set). Cell images were subjected to fluorimetric analysis in ImageJ software.

### Evaluation of β-galactosidase activity

Cells in 24-well plates (Eppendorf, No. 0030741005) were fixed with 3.7% formaldehyde (10 min, RT) and washed three times with 2% BSA solution. Detection of senescent/dormant phenotype was performed with measurement kit (CellEvent™ Senescence Green Detection Kit; Invitrogen; No. C10850; 2022) according to the manufacturer’s protocol. Subsequently, the samples were visualized with Leica DMI6000B microscope using AlexaFluor 488 filter set. The images were processed and analyzed with ImageJ software.

### Metabolic activity

For the measurements of intracellular ATP, the cells were seeded into 96-well glass bottom plates (Eppendorf; No. EP0030741030) at the density of 5 × 10^3^ cells/well, subjected to the protocol of pulse DOX treatment/regeneration, and analyzed with the ATP determination kit (Invitrogen; No. A22066) and Infinite 200 Pro Reader (Tecan) according to manufacturer’s protocol. Intracellular NAD/NADH, NADP/NADPH, α-ketoglutarate and glucose-6-phosphate content were estimated in the cells seeded into Nunc™ Cell Culture/Petri Dishes (Thermo Scientific™; No. 150350) at the density of 2 × 10^6^ cells/dish and subjected to the protocol of pulse DOX treatment/regeneration. Dedicated biochemical kits (Sigma; No. MAK037, MAK038, MAK054 and MAK014, respectively) were applied according to manufacturer protocols, followed by deproteinization (10 kDa MWCO filters; Millipore; No. UFC5010, 4^o^C, 14 000 g) and the measurements of absorbance (Multiskan™ FC Microplate Reader; ThermoFisher Scientific) at wavelengths dedicated to the individual assay. All obtained results were normalized against cell numbers (estimated with the Z2 particle counter; Beckman Coulter).

### Metabolic profiling

Metabolic phenotype of the cells was analyzed with the Seahorse XFp device according to manufacturer’s protocol. Cells were seeded into XFp-dedicated cell culture plates at density 1.5 × 10^3^/well and subjected to the protocol of pulse DOX treatment/regeneration. Directly before experiment, culture medium was replaced with DMEM (supplemented with 10 mM glucose, 1 mM pyruvate, 2 mM glutamine and 5 mM HEPES, pH = 7.4) and the cells were incubated 37^o^C/5% CO_2_ for 45 min. Their bioenergetic profile was estimated using default settings established by manufacturer (Mito Stress or Glycolysis Stress setup; 1.5 µM Oligomycin A, 1 µM FCCP, 0.5 µM Rotenone/Antymycin A, 10 mM glucose and 50 mM 2-deoxyglucose). Oxygen consumption rates (OCR) and extracellular acidification rates (ECAR) were calculated to estimate metabolic re-profiling of the cells. All obtained data were normalized against the number of cells in each culture plate well (ImageJ-assisted counting of Hoechst 33342-stained cells).

### Proteomics

#### Sample preparation for liquid chromatography and tandem mass spectrometry (LC-MS/MS)

Cells were collected in the lysis buffer (2% SDS 0,1 M Tris pH = 7,5; 3–4 biological replicates) and sonicated for 15 min. in the Bioruptor UCD-200 sonicator (Diagenode, Liege, Belgium) at 320 W (intensity: high; 30s/30s ON/OFF cycles). Then, the samples were incubated at 95^◦^C for 5 min. and centrifuged (20000 g; 10 min. in RT). Supernatants were prepared for LC-MS/MS analysis using the Filter Aided Sample Preparation (FASP) method [56]. Briefly, cell lysates were diluted in 8 M urea in 50 mM ammonium bicarbonate (300 µl), reduced with DTT (final concentration: 50 mM; 15 min.) and applied on the 30 kDa cut-off filter (Vivacon 500, Sartorius Stedim, Germany). After centrifugation (14000 g, 2 × 15 min. in RT), the proteins were washed with 200 µl of 8 M urea and centrifuged (14000 g, 2 × 45 min. in RT). The proteins were alkylated with iodoacetamide (final concentration: 0.1 mg/ml in 8 M urea, 20 min., in darkness) and the samples were washed three times with 8 M urea (14000 g, 2 × 25 min. in RT) and four times with 50 mM ammonium bicarbonate (14000 g, 2 × 20 min. in RT). After the last centrifugation step, the samples were subjected to the on-filter protein digestion (protein/trypsin ratio: 100:1 w/w in ammonium bicarbonate; overnight in 37 °C). Then, the peptides were spin down (14000 g, 30 min. in 25 °C) and the filter unit was washed two times with 40 µl of 50 mM ammonium bicarbonate and once with 50 µl of 0.5 M NaCl (14000 g; 30 min. in 25 °C). After addition of 2 µl of 100% TFA, samples were centrifuged (35000 g, 20 min. in 4 °C) and transferred into the vial inserts prior to LC-MS/MS analysis.

#### LC-MS/MS measurement

Samples were analyzed using Q Exactive high-resolution mass spectrometer (Thermo Fisher Scientific) coupled with the DPV-550 Digital PicoView nanospray source and nanoHPLC (UltiMate 3000RS LC nanoSystem; Dionex). Peptides were loaded on the C18 precolumns (Acclaim PepMap Nano trap Column; in 2% acetonitrile/0.05% TFA solution) and separated on 50 cm×75 μm RP columns (Acclaim PepMap 75 μm/100Å Nano Series™; 2–40% ACN in 0.05% FA) for 240 min. Full MS scans were acquired in the Orbitrap mass analyzer (m/z: 300–2000; resolution of 70 000 (at m/z: 200)). The top twelve most intense peaks (charge state ≥ 2) were chosen for fragmentation in the HCD collision cell (with the normalized collision energy of 27% and the isolation window of 1.2 m/z). Tandem mass spectrum was acquired in the Orbitrap mass analyser with the resolution of 17500 at m/z: 200.

#### LC-MS/MS data analysis

Raw LC-MS/MS files were analyzed using MaxQuant 2.1.4.0 software and an Andromeda server against the SwissProt database with Homo sapiens taxonomy restriction (20 404 sequences) supplemented with the common protein contaminant database. LFQ intensity and standard software settings were applied (a false discovery rate (FDR) below 1%). The search parameters were as follows: enzyme: trypsin; number of missed cleavages: 2; static modification: carbamidomethylation (C); dynamic modifications: oxidation (M) and acetyl (Protein N-term). Statistical analysis was done using Perseus software 1.6.7.0 using ANOVA permutation based FDR < 0.05 and post hoc Tukey’s tests. Protein groups from the reverse database, common protein contaminants, as well as proteins only identified by site, were filtered out (with 4897 protein groups left). Gene Ontology analysis was performed using String software (https://string-db.org; [57]).

### Quantification of ROS, GSH and lipid peroxidation

Cells seeded at the density of 2 × 10^4^ cells/well in 24-well plates (Eppendorf; GSH and lipid peroxidation assays) or in cell culture dishes (Thermo Fisher™, No. 150350; 1 × 10^6^ cells/dish; GSH/GSSG assay) were subjected to DOX (1 µM) treatment for 48 h followed by further cultivation in drug-free medium at indicated time steps. For mitochondrial ROS measurements, the cells were incubated with 2.5 µM CellROX Deep Red Reagent (Invitrogen; No. C10422) for 30 min., followed by the medium removal and the application of FluoroBrite™ DMEM supplemented with 10% FBS and 1% GlutaMAX (Gibco; No. 35050061). GSH/GSSG content and lipid peroxidation was quantified with ThiolTracker™ Violet assay (Invitrogen™; No. T10095), Glutathione GSH/GSSG Assay Kit (Sigma; No. MAK440) and Image-iT™ Lipid Peroxidation Kit (Invitrogen; No. C10445), respectively, according to the manufacturer’s protocols. Imaging was performed with the Leica Stellaris 5 microscope equipped with CO_2_ chamber (5% CO_2_) and temperature (37^o^C) monitoring system [52, 58]. Fluorimetric analyses of all acquired images were done with ImageJ software, as described above. GSH/GSSG measurements were normalized against cell numbers (estimated with the Z2 particle counter; Beckman Coulter).

### ImageStream^®^ analyses of intracellular doxorubicin and ABCB1 levels

To quantify intracellular DOX-accumulation, T98G cells were seeded into 6-well plates (Corning^®^Costar^®^) at the density of 2 × 10^4^ cells/well and incubated in the control medium for 24 h. Then, 1 µM DOX was applied with the fresh medium for the next 48 h, followed by DOX removal and further incubation of the cells for the next 3, 7 and 14 days. Then, the cells were harvested with TrypLE solution, centrifuged, resuspended in 25 µl of FluoroBrite™ DMEM medium (Gibco; No. A1896701) and analyzed in ImageStream^®^ X Mk II cytometer (Amnis) with 488 nm laser (Channel 3). For ImageStream analyses of ABCB1 levels, the cells were harvested with TrypLE solution, centrifuged, suspended, fixed with 3.7% formaldehyde and stained (as described in Sect. 2.5). Obtained data were processed in IDEAS 6.2 software (Amnis). Intracellular DOX/ABCB1 localization was visualized with Leica Stellaris 5 confocal microscope (Doxorubicin filter set).

### Statistical analysis

Unless stated otherwise (LC-MS/MS data analysis), the statistical analysis of the data was performed using two-sample t-student test (including Welch correction), non-parametric Mann-Whitney U test or one-way ANOVA with Tukey’s test for means comparison (as indicated in the legends) with Origin 2020 software (v. 9.7.0.188; OriginLab Corporation). p-values above 0.05 were considered as statistically insignificant. Tests for outliers were not performed and any datapoints were not excluded. Sample sizes correspond to those conventionally used in the in vitro analyses at the single cell and cell population level [59, 60]. Error bars illustrate ± SEM (Standard Error of the Mean) or ± SD (standard deviation) values (as indicated in the text). At least 3 biological replicates (*N* ≥ 3) have been recruited for the statistical analyses. In single-cell experiments at least 25 single cells from each experimental condition were analyzed.

## Results

### Two-phasic pattern of T98G adaptation to DOX-induced stress

**Fig. 1:**
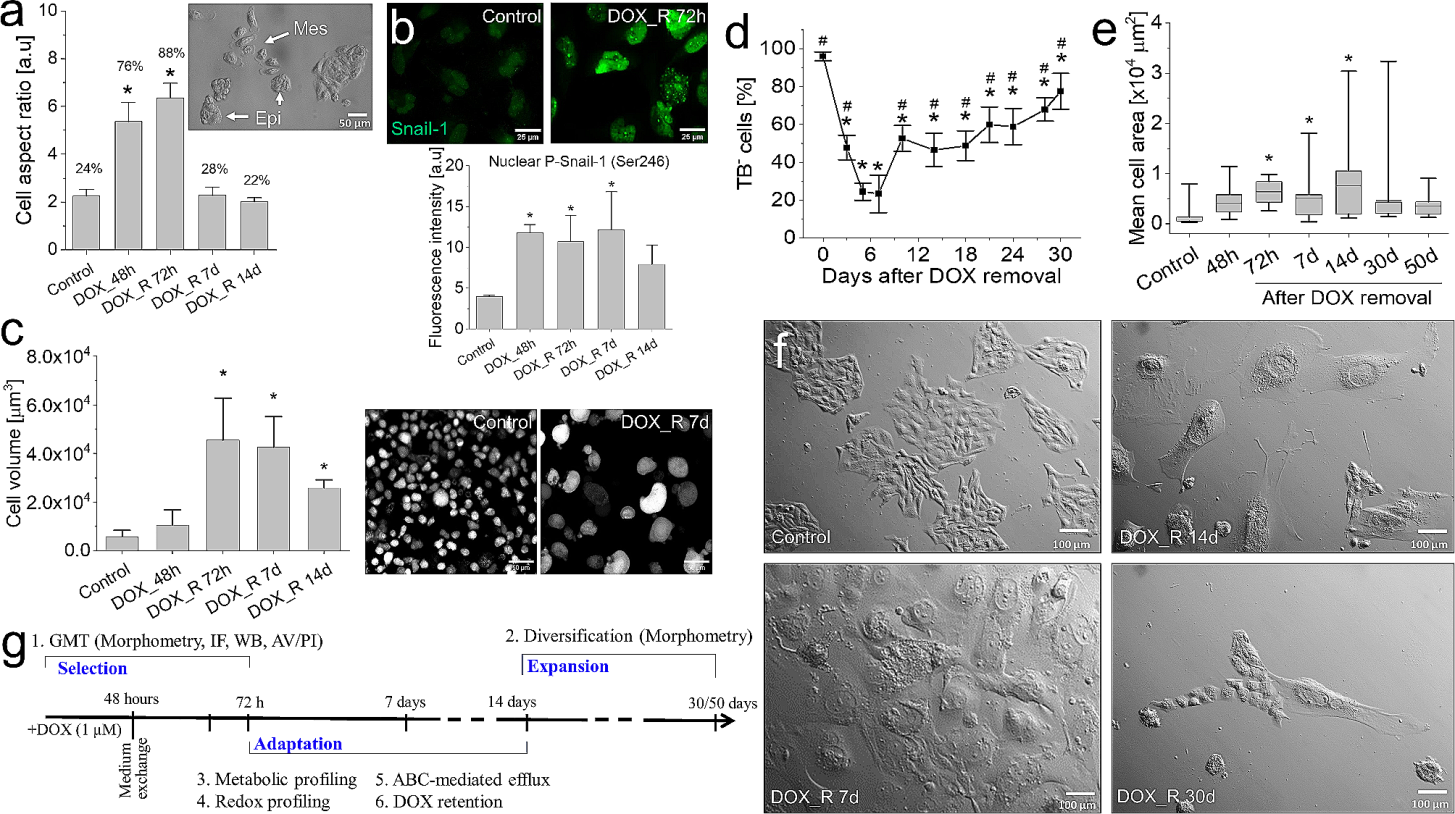
Two-phasic pattern of T98G adaptation to the pulse DOX-induced stress. (**a**) Long-term dynamics of mesenchymal cell morphology (values show % of mesenchymal cells classified according to the AR values > 2.5) following a pulse DOX-induced treatment of T98G cells, estimated with NIC microscopy-assisted morphometry (cf. Fig. S1a). (**b**) Nuclear Snail-1 (upper panel) and p-Snail(Ser246; lower panel) visualized and quantified with fluorescence microscopy and fluorimetry. (**c**) Long-term dynamics of T98G hypertrophy following a pulse DOX-induced treatment of T98G cells, estimated with volumetric approach. (**d**-**f**) The dynamics of T98G viability (left) and spreading (right) following a pulse DOX-treatment, estimated with trypan blue assay (**d**) and microscopy-assisted morphometric approach, respectively (**e**,**f**). (**g**) Experimental approach towards the identification of the processes underlying long-term GBM adaptation to the pulse DOX treatment. Scale bars = 50 μm (**a**,**c**), 25 μm (**b**) and 100 μm (**g**). Statistical significance was calculated with ANOVA and Tukey’s post hoc (**a**-**c**) and t-student test (**e**). Bars represent SEM or minimum/maximum values (**e**). Data representative for 3 independent biological replicates or ≥ 50 cells in 3 replicates. *Note* GMT of pulse DOX-treated T98G cells, followed by their long-term phenotypic diversification

Cancer microevolution is governed by the heterogeneous sensitivity of cancer cells to chemotherapeutic stress. Phenotypic heterogeneity of T98G cells was illustrated by the co-existence of non-polarized “epithelioid” and rear-front polarised “mesenchymal” clones (Fig. 1a; cf. Fig. S1a in Supplementary material), the differences in their drug-efflux efficiency (cf. Fig S1b; [61]) and their differential reactivity to the pulse DOX treatment (1 µM; 48 h). “Mesenchymal” T98G cells retained relatively high motility under DOX-induced stress, whereas the GMT and enhanced motile activity of “epithelioid” cells accounted for increased “en-mass” invasiveness of DOX-treated T98G populations (cf. Fig. S2). Long-term analyses of the consequences of pulse DOX treatment confirmed the complexity of T98G reactions to the pulse DOX treatment (1µM; 48 h). An early induction of GMT in DOX-treated T98G populations was confirmed by Snail-1 activation (Fig. 1b, cf. Fig. S3), transiently increased fractions of “mesenchymal” cells (Fig. 1a, cf. Fig. S1a and S2a) and Cx43/vimentin up-regulation (cf. Fig. S4 and S5, respectively). These events were accompanied by progressive hypertrophy of T98G cells (Fig. 1c; cf. Fig. S6) and apoptotic death (not shown) resulting in their impaired viability at 3rd -7th day after DOX removal (Fig. 1d). T98G adaptation during the 2nd phase of microevolution (7th -50th day after DOX removal) was illustrated by gradually increasing fraction of viable cells (Fig. 1d) and transient domination of viable “giant” cells around 14th day after DOX removal (characterized by projection surface areas > 5000 μm²; Fig. 1e-f, cf. Fig. S7a, b). Development of the clusters of small expanding cells (SECs) in their proximity was an ultimate step of T98G recovery from DOX-induced stress (Fig. 1f). Corresponding effects were seen in T98G populations pulse-treated with other cytostatic drugs (cf. Fig. S7c) and in pulse DOX-treated populations of U87-MG, Ln229 and Ln18 cells (cf. Fig. S8). Collectively, a two-phase scheme of phenotypic evolution underlies GBM recovery from DOX-induced stress: the short-term DOX-induced selection of GBM cells is followed by their phenotypic diversification. This observation prompted us to comprehensively trace the mechanisms underlying long-term adaptation of GBM cells to DOX-induced stress (Fig. 1g).

### Cooperative pattern of T98G recovery from DOX-induced stress

**Fig. 2:**
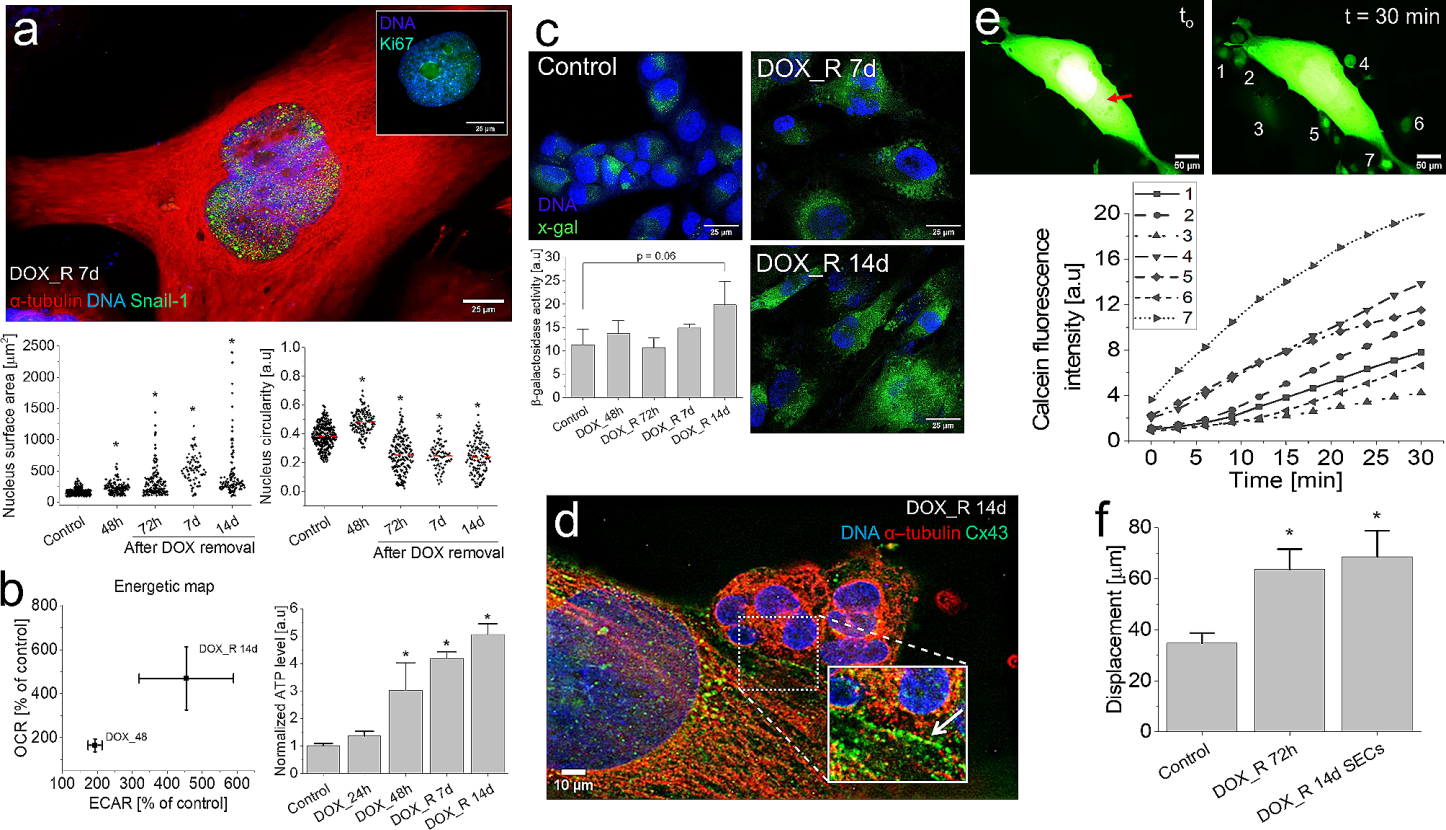
Heterogeneity of T98G populations recovering from DOX-induced stress. (**a**) Nuclear Ki67 (insert) and Snail-1 (green) localization in PGCs at the 7th day after DOX removal and the dynamics of their nuclear polymorphism following a pulse DOX-treatment. (**b**) Seahorse XFp analyses of the oxygen consumption rate (OCR) and extracellular acidification rate (ECAR, left) and ATP accumulation (right) in PGCs following a pulse DOX-treatment. (**c**) β-galactosidase activity in PGCs and SECs visualized (as x-gal fluorescence) at 14th day after DOX-removal with fluorimetry. (**d**,**e**) Cx43 gap junctions (**d**) and GJIC (**e**) between PGCs and adjacent clusters of SECs visualized with immunofluorescence and calcein microinjection/transfer, respectively. Single cells are marked according to the signature of the relevant calcein transfer curve. Red arrow indicates the dye injection site. (**f**) Motility of SECs estimated with time-lapse video-microscopy at the population level (cf. Fig. S9b in Supplementary data). Scale bar = 25 μm (**a**,**c**), 10 μm (**d**) and 50 μm (**e**). Statistical significance was calculated with ANOVA and Tukey’s post hoc test (**a**,**c**), t-student (**b**) and non-parametric Mann-Whitney test (**f**), *p < 0.05 vs. control. Error bars represent SD (**b**,**c**) and SEM (**f**). Data representative for *n* > 30 single cells and/or 3 independent biological replicates. *Note* the metabolic cooperation of dormant PGCs and SECs

“Giant” cells have long been suggested to determine the adaptation of tumor ecosystems to pharmacological stress [40, 43]. Analyses of the phenotype of “giant” cells in DOX-treated T98G populations revealed their progressive nuclear polymorphism, illustrated by increasing fractions of relatively big, non-circular nuclei (> 250 µm^2^; circularity < 0.25) between the 3rd and 14th day after DOX removal (Fig. 2a). It was accompanied by relatively high values of OCR/ECAR and ATP accumulation (Fig. 2b) in T98G populations. Together with the gradual induction of β-galactosidase activity (Fig. 2c) and the nuclear localization of Ki67 and Snail-1 in these cells (Fig. 2a), these observations demonstrate the dormant phenotype of DOX-induced poly(morpho)nuclear giant cells (PGCs) [38–40]. Their significance for the DOX-induced microevolution of GBM populations was illustrated by the signs of metabolic cooperation between PGCs and SECs. It is manifested by their prevalent proximity and the development of abundant Cx43^+^ gap junctions and microvilli at PGC/SEC interfaces following the 14th day after DOX removal (Fig. 2d; Fig. S9a). Calcein transfer assay revealed high efficiency of gap junctional intercellular coupling (GJIC) between calcein-microinjected PGCs and the adjacent SECs (Fig. 2e), confirming the metabolic cooperation of PGCs and SECs. In turn, the welfare of SECs was demonstrated by their relatively high motility (Fig. 2f, cf. Fig. S9b). Collectively, long-term viability of dormant PGCs and their metabolic cooperation with SECs indicate the active “chaperon” function of PGCs, which underlies the early stages of GBM adaptation to the DOX-induced stress.

### Metabolic background of DOX-induced PGC program

**Fig. 3:**
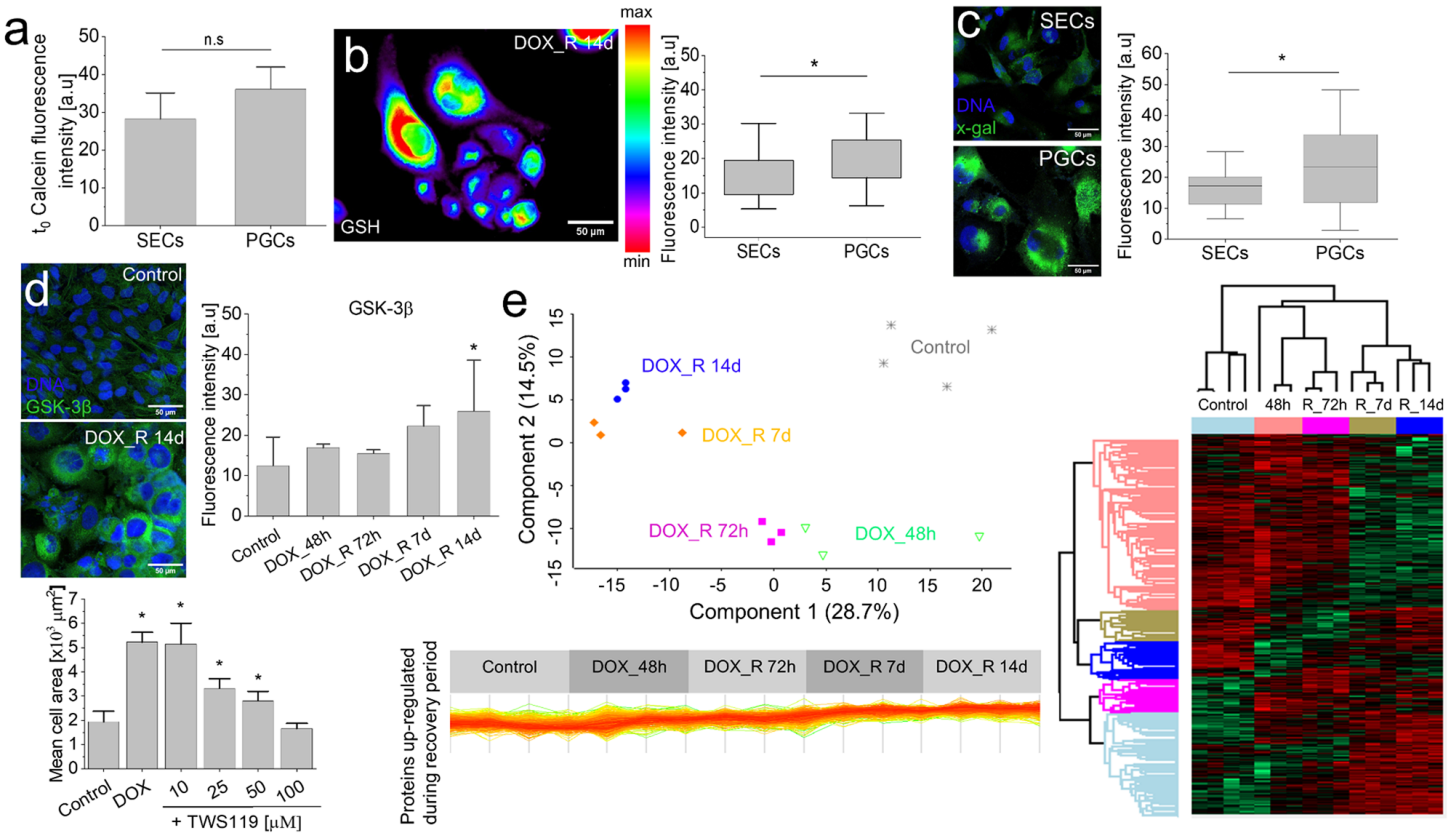
Hallmarks of DOX-induced PGC program. (**a**) Drug-efflux efficiency in PGCs and SECs cells estimated with calcein efflux assay. (**b**) GSH levels in PGCs and SECs following the pulse DOX-treatment. (**c**) β-galactosidase activity in PGCs estimated with x-gal assay. (**d**) GSK-3β levels in DOX-induced PGCs (upper panel) and the effect of TWS119 on the efficiency of DOX-induced PGC formation (lower panel). (**e**) Variance analysis of T98G proteomes following a pulse DOX treatment (upper left panel), proteomic map of up- (red) and down-regulated (green) proteins (right panel) and the cluster of proteins up-regulated after DOX removal (lower left panel, cf. Fig. S11). Scale bars = 50 μm. Error bars represent SEM values. Statistical significance was calculated with t-student (**a**-**c**) or ANOVA and Tukey’s post hoc test (**d**), *p < 0.05 vs. control. Data representative for > 50 cells and/or 3 independent biological replicates. *Note* the high metabolic activity and extensive proteomic reprogramming of PGCs

Protective potential of PGCs primarily depends on the efficiency of their self-defense reactions. Therefore, further analyses were focused on the activity of stress management systems in adherent T98G populations at the 14th day after DOX-removal, i.e. at the time-point of maximal abundance of viable PGCs (cf. Figures 1e-g and 2a and b). These analyses revealed a relatively high activity of ABC transporters (Fig. 3a), GSH levels (Fig. 3b) and β-galactosidase activity in PGCs (in comparison to SECs; Fig. 3c). A coordinated activation of these systems was accompanied by the up-regulation of glycogen synthase kinase-3β in DOX-induced PGCs (Fig. 3d, cf. Fig. S10). The attenuation of PGC formation and SEC expansion, which was observed after the application of chemical GSK-3β inhibitor (TWS119), suggests that GSK-3β-dependent adaptive phenotypic program underlies the DOX-induced PGC formation. Also LC-MS/MS analyses revealed extensive proteomic reprogramming (cf. Fig. S11a) illustrated by five multiprotein clusters, distinguished according to the pattern of the protein up/down-regulation (cf. Fig. S11b). Notably, the cluster of constantly up-regulated proteins comprises β-galactosidase (cf. Fig. S1c) and metabolic enzymes (cf. Figure 3e; Appendix 1). These proteomic data, along with progressive decrease of the inter-sample variances between 7th and 14th day after DOX removal (Fig. 3e) and the metabolic mobilization of PGCs (cf. Figure 2b), indicate the metabolic background of the DOX-induced, GSK-3β-dependent, adaptive PGC program. This notion prompted us to focus (i) on the dynamics of the metabolic PGC profile and (ii) on its links with the capability of PGCs to manage the DOX-induced stress.

### Dynamics of metabolic profile of PGCs following pulse DOX treatment

**Fig. 4:**
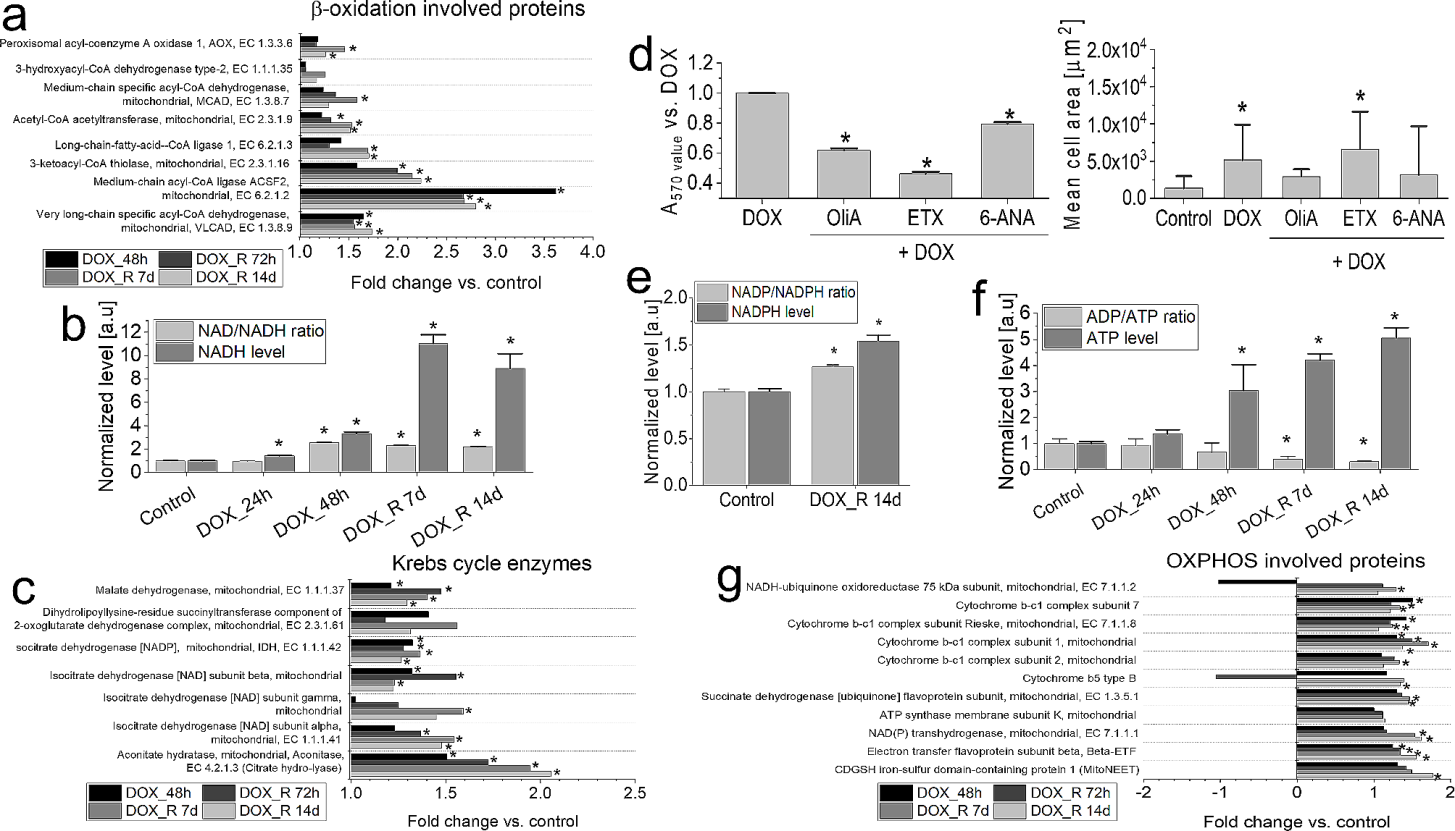
DOX-induced metabolic reprogramming of T98G cells. (**a**,**c**,**g**) Up-regulated β-oxidation (**a**), Krebs cycle proteins (**c**) and mitochondrial respiration proteins (**g**; cf. Fig. S12 for their STRING-generated interactomes) revealed by LC-MS/MS proteomic approach. (**b**,**f**) Intracellular NADH/NAD (**b**) and ATP/ADP levels in T98G cells (**f**) analysed at the indicated time-points following a pulse DOX treatment. (**d**) The effect of oligomycin A (OliA; 5µM), etomoxir (ETX; 250 µM) and 6-ANA (5µM) on the metabolic activity (left) and PGC formation (right) in T98G populations following a pulse DOX-treatment. (**e**) Quantification of intracellular NADP/NADPH levels in T98G cells at 14th day following a pulse DOX treatment. Statistical significance of the differences was calculated with ANOVA permutation based FDR < 0.05 and post hoc Tukey’s test (**a**,**c**,**g**), ANOVA and Tukey’s post hoc test (**d**, left) and t-student test (**b**; **d**,right, **e**,**f**), *p < 0.05 vs. control. Bars represent SD values. Data representative for >50 cells or 3 independent biological replicates. *Note* the sensitivity of PGC program to PPP and OXPHOS inhibitors, accompanied by a prominent NADH/ATP and negligible NADPH accumulation in PGCs

Proteomic approach was applied to identify the hallmarks of metabolic reprogramming and its significance for PGC program in pulse DOX-treated GBM cells. These studies revealed the up-regulation of the enzymes responsible for the mitochondrial β-oxidation in PGCs (Fig. 4a, cf. Fig. S12 and Appendix 1). It was accompanied by the activation of the systems managing ATP/ADP homeostasis, unsaturated fatty acids oxidation and carnitine transport (Fig. S13a), and the prominent accumulation of NADH and the elevated NAD resources in PGCs (Fig. 4b). Krebs cycle contribution to NADH generation in PGCs was demonstrated by the mobilization of Krebs cycle enzymes (Fig. 4c), in the absence of the inhibitory effects of etomoxir (ETX; β-oxidation inhibitor) on PGC formation (Fig. 4d). NADH accumulation in PGCs was paralleled by the attenuation of DOX-induced PGC program and eradication of clustered SECs upon the chemical inhibition of pentose phosphate pathway (PPP; by 6-aminonicotinamide; 6-ANA) and oxidative phosphorylation (OXPHOS; by oligomycin A (OliA; Fig. 4d, cf. Fig S13b). These observations illustrate the significance of NADPH and ATP producing pathways for PGC program. On the other hand, only a negligible NADP and NADPH accumulation was detected in these cells (Fig. 4e). In turn, a distinct ATP accumulation and decreased ADP/ATP ratios in GBM cells (Fig. 4f, cf. Fig. S13c) was accompanied by the up-regulation of the enzymes involved in the oxidative phosphorylation (OXPHOS), incl. the subunits of the cytochrome bc_1_ complex, NADH-ubiquinone oxidoreductase and ATP synthase (Fig. 4g). These data confirm the metabolic background of PGC program in pulse DOX-treated GBM populations.

### Oxidative stress management in DOX-loaded PGCs: the activation of ROS scavengers

**Fig. 5:**
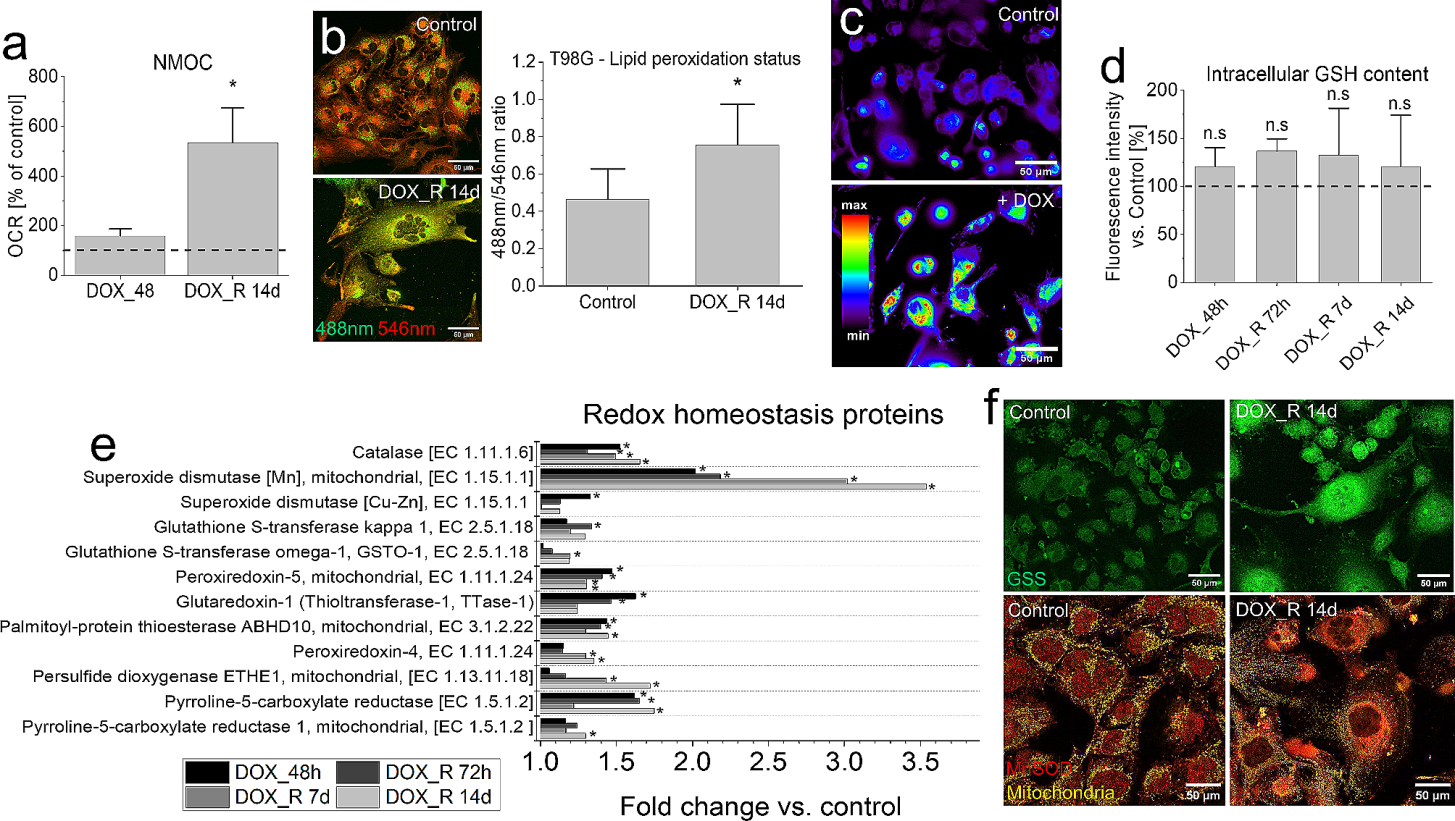
ROS scavengers in DOX-induced PGCs. (**a**) Seahorse XFp analyses of the short- and long-term DOX effects on the non-mitochondrial oxygen consumption (NMOC) in T98G cells. (**b**,**c**) Lipid peroxidation status (analyzed with Image iT™ Lipid Peroxidation kit, (**b**) and ROS levels (**c**) estimated 14 days after DOX removal and 6 h after DOX application, respectively. (**d**) Intracellular GSH content estimated with the fluorescence microscopy-assisted (ThiolTracker™ Violet assay; Spectrum LUT mask) at the indicated time-points. (**e**) The cluster of ROS managing proteins revealed by LC-MS/MS proteomic analyses (cf. Fig. S12 for their STRING-generated interactome). (**f**) Localization of MnSOD and glutathione synthetase (GSS) in DOX-induced PGCs. Scale bars = 50 μm. Statistical significance was calculated with t-student test (**a**,**b**,**d**) or ANOVA permutation based FDR < 0.05 and post hoc Tukey’s test (**e**), *p < 0.05 vs. control. Data representative for 4 independent biological replicates (random microphotographs; **b**,**c**,**f**). Error bars represent SD. *Note* low levels of oxidative stress in PGCs accompanied by the mobilization of mitochondrial and cytoplasmic ROS scavenging systems

Further experiments were performed to elucidate the links between the selective accumulation of electron carriers in PGCs and efficiency of their stress management systems. Because DOX cytotoxicity is related to the generation of oxidative stress, negligible NADPH accumulation in PGCs (cf. Figure 4e) can result from the high NADPH consumption. Pro-oxidative activity of DOX in PGCs was confirmed by the relatively high non-mitochondrial O_2_ consumption rates (NMOC) in these cells, which reached > 500% of the control values (Fig. 5a). Concomitantly, the signs of oxidative stress and the mobilization of ROS scavenging systems were demonstrated by the moderate elevation of lipid peroxidation (Fig. 5b), ROS (Fig. 5c), GSH and GSH/GSSG ratio in DOX-intoxicated T98G (Fig. 5d, cf. Fig. S14a, b), U87-MG, Ln18 and Ln229 PGCs (cf. Fig. S15a, b). The mobilization of detoxification systems in PGCs was also confirmed by proteomic studies. They distinguished the cluster of ROS scavenging enzymes that were considerably up-regulated in T98G PGCs between 7th and 14th day after DOX removal (Fig. 5e, cf. Fig. S14c and Appendix 1). It comprised catalase and superoxide dismutase (i.e. the enzymes that neutralize H_2_O_2_ and its products) as well as NADPH- and GSH-dependent ROS scavengers (incl. reductases and S-transferases, respectively) and glutathione synthetase (GSS; Fig. 5f). Collectively, these observations confirm that the activation of NADPH/GSH-dependent ROS scavenging systems accounts for the longevity and DOX-resistance of PGCs and for the negligible NADPH accumulation in these cells.

### Oxidative stress management in DOX-loaded PGCs: the fine-tuning of ROS production

**Fig. 6:**
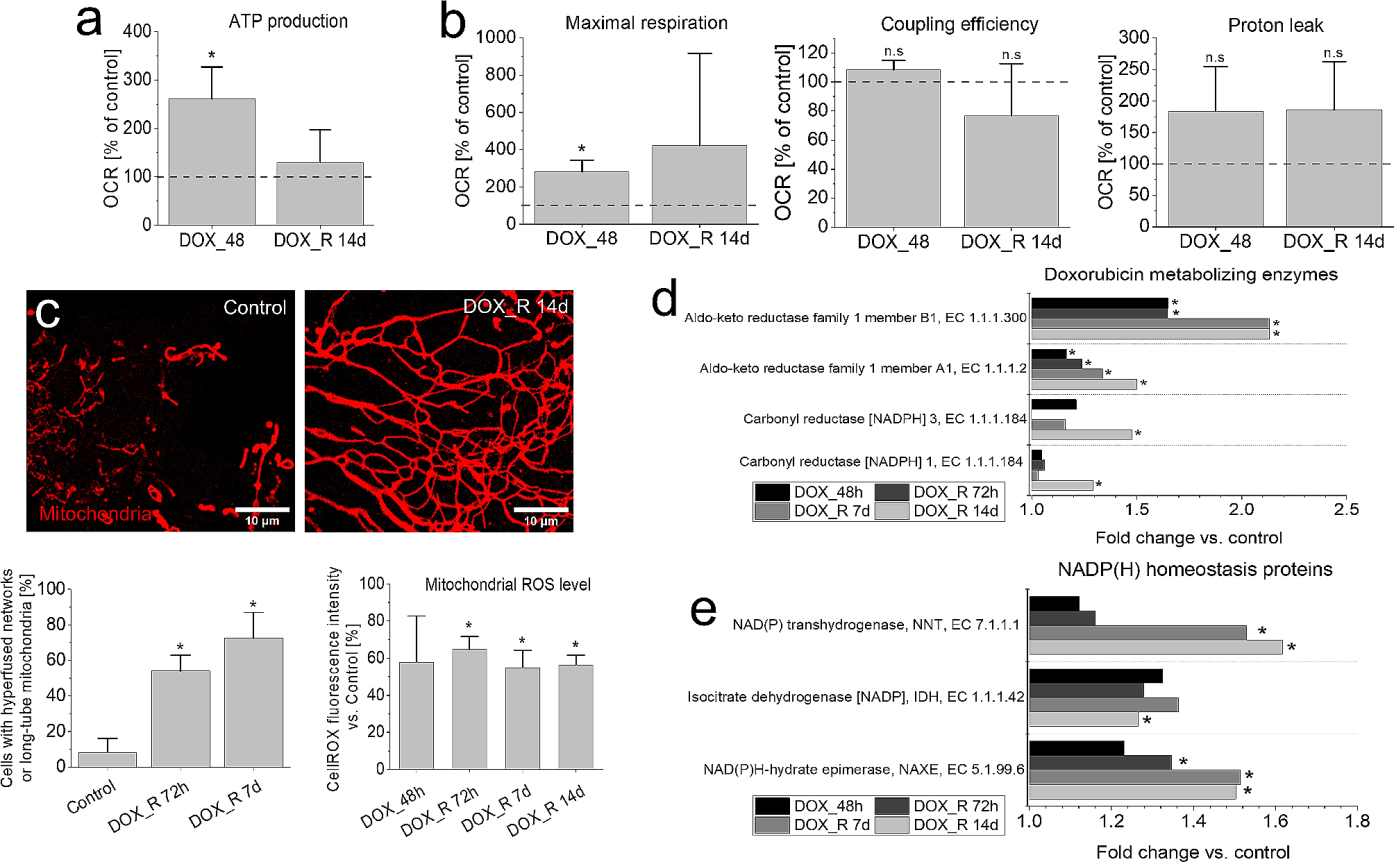
Management of ROS production in PGCs. (**a**,**b**) Seahorse XFp analyses of the short- and long-term DOX effects on the mitochondrial ATP production (**a**) and on the integrity of mitochondrial respiration system in T98G cells (**b**). (**c**) Morphology of PGC mitochondria (left, middle) and mitochondrial ROS levels (right) estimated with fluorescence microscopy-assisted CellROX DeepRed dye assay. (**d**,**e**) The clusters of up-regulated proteins involved in the in DOX inactivation/metabolism (**d**) and NAD/NADP homeostasis (**e**) estimated with LC-MS/MS proteomic approach. Scale bars = 10 μm. Statistical significance was calculated with t-student test (**a**,**b**,**c**:mitofusion), ANOVA and Tukey’s post hoc test (**c**:ROS) or ANOVA permutation based FDR < 0.05 and post hoc Tukey’s test (**d**,**e**), *p < 0.05 vs. control. Bars represent SD, from 3 independent biological replicates in all panels. *Note* the fine-tuning of mitochondrial stress, accompanied by the mobilization of NADH/NADPH conversion and NADPH-dependent DOX degradation system

Oxidative stress results from intracellular disbalance between ROS scavenging and production. Return of OXPHOS intensity in DOX-intoxicated PGCs (visualized by ATP production) to the control values (Fig. 6a), accompanied by gradual NADH accumulation (cf. Figure 4b), suggested a strict control of both processes in these cells. It was further confirmed by increased respiration capacity of the mitochondria, a negligible proton leak (Fig. 6b) and relatively low ROS levels in the mitochondrial networks (Fig. 6c, cf. Fig. S16a). Concomitant activation of glycolysis and Krebs cycle (cf. Fig. S16b, c) ultimately demonstrated the absence of NADH-dependent reductive stress in PGCs. Intensified NMOC and glycolysis (ECAR), accompanied by the increased respiration capacity and mitochondrial fusion, was also observed in 100 nM DOX-induced U87-MG PGCs (cf. Fig. S17a-d).

However, increased respiration parameters and proton leak in these cells indicates their less pronounced metabolic plasticity, even if the clusters of SECs were still generated in U87-MG populations (cf. Fig. S17d). In DOX-treated T98G PGCs, NADH accumulation was accompanied by the up-regulation of NADPH-dependent enzymes involved in the reductive DOX inactivation (incl. aldo-keto reductases (AKR) and carbonyl reductases; CBR; Fig. 6d, Appendix 1) [62, 63]. Furthermore, proteomic studies revealed the mobilization of the enzymes participating in NADH/NADPH conversion and NAD(P) homeostasis, incl. NAD(P) transhydrogenase (NNT), isocitrate dehydrogenase (IDH) and NAD(P)HX epimerase (NAXE; Fig. 6e). Thus, an auxiliary NNT-mediated NADH/NADPH conversion system may finely tune the NADPH levels in PGCs to limit the generation of hydroxyl radicals during the NADPH-dependent DOX degradation, while sustaining the activity of DOX degradation and ROS scavenging systems (cf. Figure 5) [62, 63]. Negligible effects of extrinsic ROS scavengers (NAC, ASC and taurine) on the DOX-induced PGC formation showed that PGC program was not activated by oxidative stress (cf. Fig. S18). They also confirm the efficiency of ROS management system in PGCs. Collectively, a balanced NADH production and NADH/NADPH conversion finely tunes intracellular NADPH bioavailability in PGCs. It synchronizes NADPH/DOX-dependent ROS production with the activity of ROS scavenging systems to effectively manage the DOX-induced stress.

### DOX retention in PGCs

**Fig. 7:**
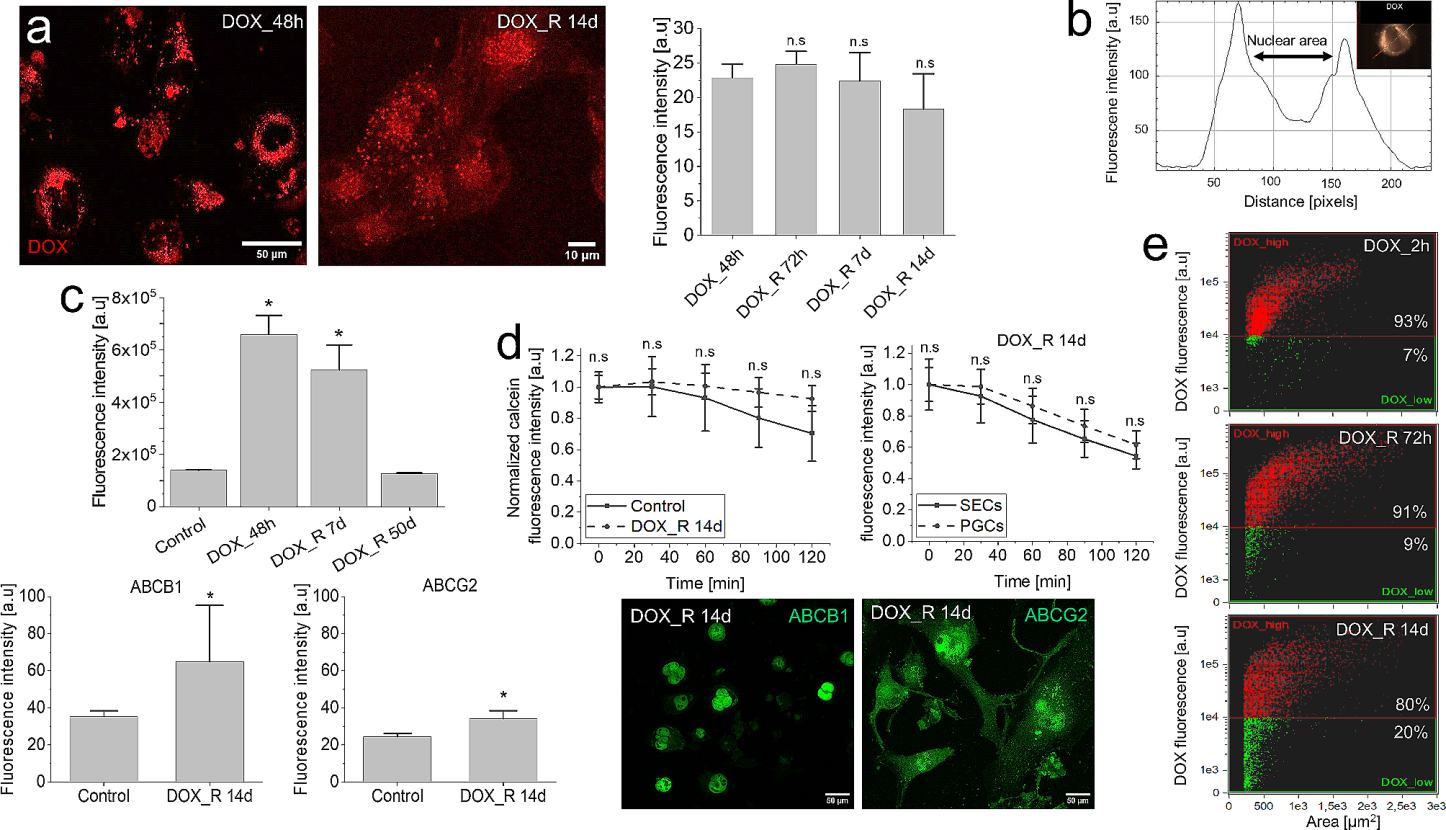
DOX retention in PGCs. (**a**) Intracellular DOX retention in PGCs following a pulse DOX treatment. DOX-specific fluorescence was registered at the indicated time-points and quantified with immunofluorescence and fluorimetry. (**b**) Perinuclear DOX accumulation in PGCs estimated with ImageStream^®^flow-cytometer. (**c**) ImageStream^®^ (upper panel) and fluorimetric analyses of ABCB1 and ABCG2 levels (lower panel) in T98G cells following their a pulse DOX-treatment. (**d**) Calcein efflux efficiency in PGCs (upper panel) and perinuclear ABCB1/ABCG2 accumulation in PGCs (lower panel) estimated with calcein efflux (vs. control cells (upper left) and SECs (upper right)) and immunofluorescence, respectively. (**e**) A progressive formation of DOXlow SECs in pulse DOX-treated T98G populations visualized by ImageStream. Scale bars = 50 (**a; left**,**d**) and 10 μm (a; right). Statistical significance of the differences was calculated with t-student test, *p < 0.05 vs. control. Data representative for > 50 cells (**a**,**c**; lower panels, **d**) and > 800 single cells in 3 independent biological replicates (**c**; upper panel, **e**). Error bars represent SD (**a**,**d**) or SEM (**c**) values. *Note* the expansion of DOXlow population(s) following the DOX retention/compartmentation in PGCs

Final analyses were performed to identify the links between metabolic reprogramming, DOX-induced stress management and the chaperon functions of PGCs. The presence of a DOX^high^ cell population in PGCs for up to the 14th day after DOX removal demonstrated their long-term DOX retention capacity (Fig. 7a). It was accompanied by nuclear DOX extrusion and its largely cytoplasmic localization in PGCs, which suggested the activation of perinuclear ATP-dependent intracellular DOX transport systems (Fig. 7b; cf. Fig. S19a-b).

Moreover, transient up-regulation of ABCB1 followed the pulse DOX-treatment of T98G cells (Fig. 7c). This effect was observable immediately after DOX administration and persisted till the 14th day after DOX removal. Surprisingly, no significant increase of calcein efflux efficiency in DOX-treated PGCs could be seen (Fig. 7d), whereas largely (peri)nuclear accumulation of ABCB1 and ABCG2 in PGCs was demonstrated by fluorescence microscopy. In conjunction with the longevity of PGCs (Fig. 1f), these data indicate that ABCB1/ABCG2-dependent cytoplasmic DOX compartmentalization in PGCs limits its genotoxicity. The significance of this process for the welfare of SECs was illustrated by the expansion of DOX_low_ cells (from 7 to 20%; Fig. 7e) following the PGC formation. Notably, low drug-efflux capacity of U87-MG cells (cf. Fig. S19c) correlated with the negligible mobilization of ABC transporters under 1 µM DOX stress (cf. Fig. S19d), considerable nuclear accumulation of DOX in these cells and pronounced cytotoxic DOX effects (cf. Fig. S19e-f). Collectively, ATP accumulation in PGCs and the activity of ATP-dependent drug-efflux systems facilitate the protective intracellular DOX compartmentalization and retention in PGCs. Together with the metabolic reprogramming and the activation of NADH/NADPH-dependent detoxification systems, these processes constitute the functional core of a long-term, adaptive PGC program in GBM populations.

## Discussion

**Fig. 8:**
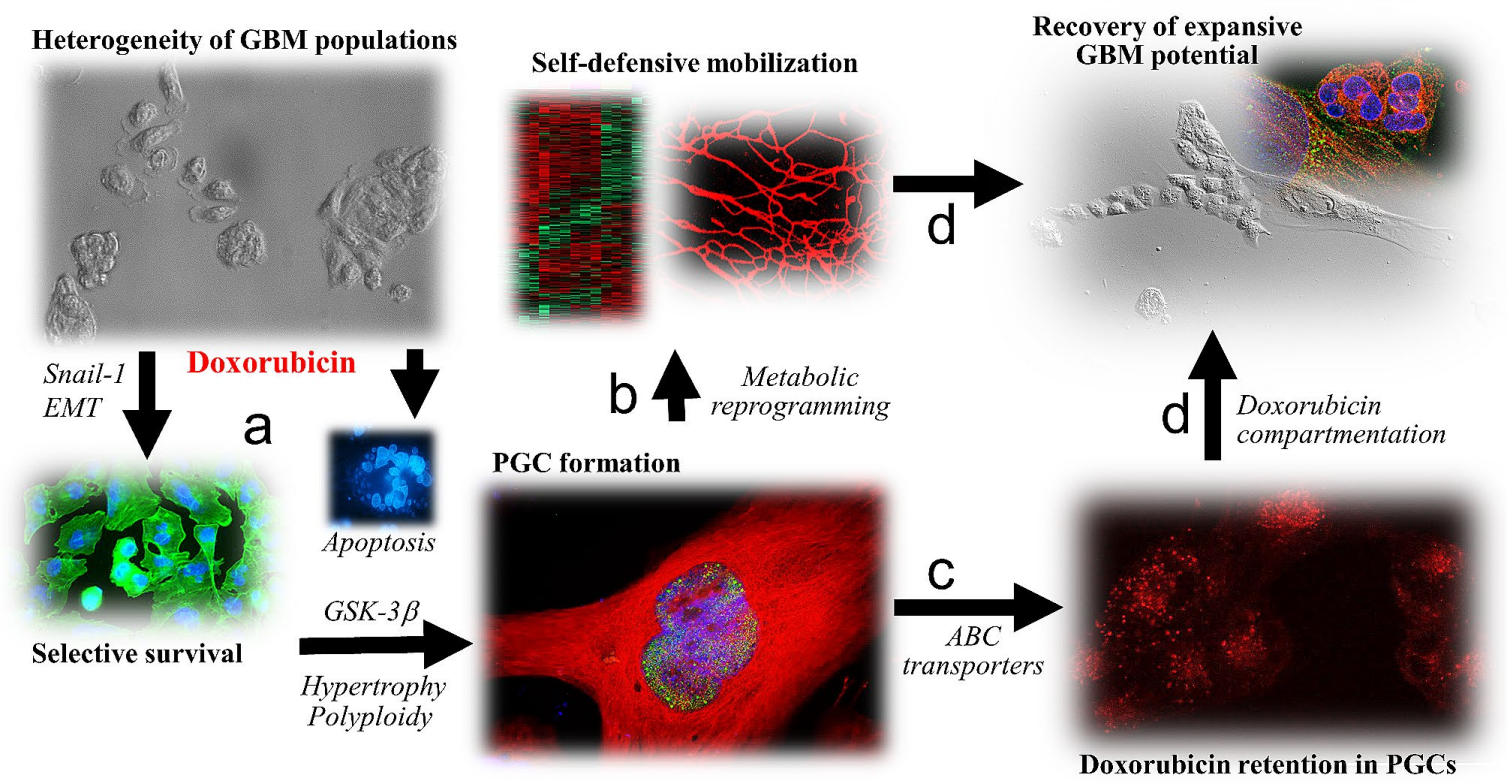
Cooperative adaptation of GBM cells to DOX-induced stress. Differential drug-resistance of GBM cells results in the selective extinction of drug-sensitive cells and GMT-related adaptation of their drug-resistant counterparts (**a**) that result in the transient formation of PGCs. Their pro-oxidative metabolic reprogramming (**b**) provides the energy resources for the mobilized self-defense and drug-retention systems (**c**). Thus, PGCs provide a protective microenvironment for small expanding cells (SECs), securing GBM recovery from DOX-induced stress (**d**)

Chemotherapy-induced microevolution of cancer drug-resistance is determined by the “selective elimination” of drug-sensitive cells and the adaptation of their more resistant counterparts to chemotherapeutic stress [64]. These processes cooperate to trigger the “selective expansion” of drug-resistant cell lineages that recolonize tumor niches after the cessation of chemotherapy [9, 10, 30, 35, 60, 61]. Despite the therapeutic significance of long-term cancer adaptation to chemotherapeutic stress, the research on this topic is predominantly focused on the short-term cytotoxic effects of anti-cancer drugs (incl. doxorubicin). In particular, cooperation between discrete cancer cell lineages during their adaptation to the chemotherapeutic stress is commonly overlooked, even though the cooperative pattern of drug-induced cancer microevolution has been suggested [64–66]. In this study, the long-term phenotypic, proteomic and metabolic profiling was combined to show the cooperative adaptation of GBM cells to DOX-induced stress. In the 1st phase of this process, differential drug-resistance of the cells underlies alternative scenarios of (i) selective extinction of drug-sensitive cells and (ii) the development of PGCs from drug-resistant cells (Fig. 8a). In its 2nd phase, metabolic reprogramming of PGCs (Fig. 8b) facilitates the mobilization of self-defense systems and their long-term DOX retention capacity (Fig. 8c). Thus, PGCs provide a protective microenvironment for small expanding cells (SECs), securing GBM recovery from DOX-induced stress (Fig. 8d).

Consecutive peaks of selective cell death, polyploidy, and cell expansion have long been suggested to delineate the drug-induced cancer microevolution [64, 67, 68]. Moreover, glial-mesenchymal transition (GMT) has been found to account for differential drug-resistance of epithelioid and mesenchymal (fibroblastoid) GBM cell sub-lineages [61, 69, 70]. In pulse DOX treated T98G populations, it underlies the preferential elimination of DOX-sensitive lineages and the recruitment of their more resistant post-GMT counterparts to the PGC program. Short-term induction of Snail-1/Cx43^high^ phenotype remains in concordance with the previously reported involvement of Snail-1/Cx43-dependent signaling axis in pro-invasive transitions of cancer cells [55]. However, subsequent development of poly(morpho)nuclear giant cells (PGCs), followed by formation of SEC clusters in a range of model GBM cell lines indicates that GMT is a preliminary stage rather than the ultimate outcome of GBM adaptation to DOX-induced stress. Nuclear localization of Ki67 and Snail-1 in PGCs, accompanied by elevated β-galactosidase levels in these cells indicates the state of their “reversible senescence” [71, 72]. Accordingly, PGCs can participate in the neosis (budding) of diploid cells (SECs) and/or fulfill protective functions in DOX-intoxicated GBM [38–41]. The generative PGC function needs to be verified. However, the significance of PGCs for the welfare of SECs in DOX-intoxicated GBM populations is confirmed by their metabolic cooperation with SECs, “solidary” extinction of PGCs and SECs after the application of metabolic blockers and chemical GSK-3b inhibition, and corresponding scenarios of consecutive cell hypertrophy and expansion in other model GBM cell lines. Conceivably, remarkable metabolic activity and the efficiency of stress-management/DOX retention systems in PGCs underlies their protective role in GBM recovery from DOX-induced stress [39, 42].

Efficient stress-management in cancer cells relies on the coordinated activity of cell detoxication and drug-inactivation systems [37], which depends on the energy supply. Metabolic mobilization of PGCs in DOX-treated GBM populations is illustrated by the up-regulation of the enzymes involved in the β-oxidation, Krebs cycle and OXPHOS. It was followed by intensified OXPHOS and glycolysis that resulted in the accumulation of NADH and ATP in PGCs. Universal significance of the accumulation of energy resources for DOX-induced GBM microevolution was additionally supported by a corresponding phenomenon observed in DOX-induced U87-MG, Ln229 and Ln18 PGCs. Ultimate extinction of GBM populations after the chemical OXPHOS inhibition confirms the significance of pro-oxidative metabolic reprogramming for the self-protective and “chaperon” potential of PGCs. Pro-oxidative metabolic activation of cancer cells under chemotherapeutic stress has been observed before [36, 45, 47, 49, 50, 73]; however this is the first report that demonstrates this process in PGCs. GSK-3β up-regulation in DOX-treated T98G PGCs and their sensitivity to chemical GSK-3β inhibition suggests the involvement of this kinase in metabolic PGC reprogramming. Corresponding “signaling hub” functions of GSK-3β have been revealed in other models, where GSK-3β regulated cell proliferation, quiescence and invasiveness, coordinating microenvironmental adaptation of cancer cells with their metabolism [74, 75]. On the other hand, DOX-induced oxidative stress was not responsible for the initiation of PGC program, as demonstrated by the lack of inhibitory effect of ROS scavenging on DOX-induced PGC formation. Collectively, PGC formation is facilitated by GSK-3β-regulated metabolic reprogramming, which prompts the mobilization of the self-defense systems in these cells, secures their welfare and facilitates their “chaperon” functions.

The mobilization of detoxification systems in DOX-induced PGCs was illustrated by the up-regulation of mitochondrial and cytoplasmic ROS scavenging enzymes, the maintenance of GSH pool and increased GSH/GSSG ratio, low mitochondrial ROS and moderate lipid peroxidation levels in these cells. The lack of the interference of extrinsic ROS scavengers (NAC, Tau and Asc) with the DOX-induced PGC formation confirmed an effective ROS management, even though increased lipid peroxidation suggests a certain level of oxidative stress in PGCs. It can be managed by the adaptive control of ROS scavenging and production, as illustrated by differential NADPH and NADH accumulation in PGCs. Negligible accumulation of NADPH in PGCs (and their sensitivity to PPP inhibition) can be explained by the activity (and significance) of NADPH-dependent ROS scavenging- and reductive DOX-degradation systems [76, 77]. Apart from their direct protective function, these systems can facilitate the strict control of NADPH levels in PGCs, limiting NADPH-dependent generation of hydroxyl radicals by DOX-degradation systems. The fine-tuning of NADPH bioavailability in DOX-intoxicated PGCs can be additionally secured by the mobilization of NNT-dependent NADH/NADPH conversion/shuttling system, the mobilization of enzymes involved in NADPH homeostasis (NAXE and IDH) and the mobilization of NADH generating pathways in PGCs (incl. glycolysis, Krebs cycle and β-oxidation). Together with the progressive inhibition of OXPHOS-related NADH consumption in PGCs, these processes represent the core of the auxiliary adaptive system in PGCs that cooperates with ROS scavenging and DOX-degrading systems to limit the pro-oxidative DOX activity. Collectively, a fine-tuning of aerobic and glycolytic ATP production cooperates with effective ROS management in DOX-intoxicated PGCs to facilitate their “chaperon” function.

The rational for permanent mobilization of ROS management systems in the PGCs is provided by the long-term DOX compartmentalization and retention in these cells. Perinuclear accumulation of DOX and ABC transporters indicates that these transporters participate in DOX relocation from PGC nuclei. On the other hand, nuclear DOX accumulation in U87-MG cells, followed by ultimate extinction of their populations after pulse DOX treatment, can be ascribed to negligible mobilization of ABC transporters in these cells. Till now, drug compartmentalization and retention in cancer cells have not received adequate attention. Our data show that ABC transporters limit the genotoxic activity of DOX by the compartmentalization of this drug in the cytoplasmic domains of PGCs [78, 79]. Moreover, DOX retention and the re-uptake of DOX released from dying cells reduce its microenvironmental bioavailability. Thereby, it mitigates the local cytotoxic DOX “tsunami”, supporting GBM recovery from chemotherapeutic stress. Energy demand of DOX retention systems justifies the synchronization of PGC metabolism with self-defense systems. Accordingly, the metabolic plasticity of PGCs participates in the mobilization of ROS scavenging/DOX management systems, facilitating their adaptation to the limited niche resources.

## Conclusions

Collectively, long-term analyses of GBM cell responses to cytostatic drugs enabled us to describe a cooperative scenario of GBM regeneration following DOX treatment. They also demonstrated a crucial role of PGCs and their metabolic reprogramming in this process. Metabolic reprogramming enhances the efficiency of self-defence systems and increases DOX retention capacity of PGCs, potentially reducing DOX bioavailability in the proximity of SECs. At the tumor tissue level, the cooperation of discrete dormant and expanding cell lineages in discrete GBM compartments can govern diverse patterns of GBM adaptation to DOX-induced stress. PGCs act to “serve and protect” expansive GBM lineages that can further colonize DOX^low^ niches and prompt the invasive GBM relapses [42, 43]. At the single-cell level, the chaperon functions of PGCs rely on their DOX retention capability. At the sub-cellular level, the long-term adaptive PGC program secures the balance between DOX compartmentation, its retention and the management of DOX-induced oxidative stress [9, 51]. This program involves metabolic reprogramming of PGCs that finely tunes the balance between the DOX inactivation and DOX-induced oxidative stress. It remains to be elucidated, whether functionally and spatially integrated intracellular “DOX-management hubs” coordinate these processes. Also the “generative” role of PGC (i.e., the neosis of SECs) requires further studies [80]. Finally, it is unclear whether the cooperation of SECs with PGCs can participate in their “stem-like” reprogramming in vitro and in vivo [41, 54]. However, DOX-induced phenotypic microevolution of GBM has already been observed in vitro and in vivo [81, 82]. Similarly, PGCs have been implicated in GBM development [39, 42, 83]. Our data give the insight into the interrelations between DOX-induced PGC formation and their metabolic reprogramming as a prerequisite for DOX-induced GBM microevolution.

### Electronic supplementary material

Below is the link to the electronic supplementary material.

Supplementary Material 1

Supplementary Material 2

## Data Availability

The data that supports the findings of this study are available in the supplementary material of this article or available from the corresponding author upon reasonable request.

## Abbreviations

ABC: ATP-binding cassette
ABCB1: ATP-binding cassette sub-family B member 1
ABCC1: ATP-binding cassette sub-family C member 1
ABCG2: ATP-binding cassette sub-family G member 2
ATCC: American Type Culture Collection
ATP: Adenosine triphosphate
Cx43: Connexin43
DMEM: Dulbecco’s Modified Eagle Medium
DMSO: Dimethyl sulfoxide
DOX: Doxorubicin
EMT: Epithelial-mesenchymal transition
FBS: Fetal bovine serum
GBM: Glioblastoma multiforme
GMT: Glial-mesenchymal transition
GSK-3: Glycogen synthase-3beta
HRP: Horseradish peroxidase
IMC: Integrated modulation contrast (Hoffman contrast)
PBS: Phosphate buffered saline
PGCs: Polyploid giant cells
PVDF: Polyvinylidene difluoride
TEM: Transmission electron microscopy
TIRF: Total internal reflection fluorescence
TMI: Transmigration index
TMZ: Temozolomide
