# Supplementary material for: Metabolic reprogramming of poly(morpho)nuclear giant cells determines glioblastoma recovery from doxorubicin-induced stress
Source: J Transl Med. 2024 Aug 12;22:757. doi: 10.1186/s12967-024-05541-9 (PMC11318163; doi:10.1186/s12967-024-05541-9)
Supplement: Supplementary file 1 — Supplementary Material 1 [file 12967_2024_5541_MOESM1_ESM.docx]

*Supplementary data*

**Metabolic reprogramming of poly(morpho)nuclear giant cells determines**

**glioblastoma recovery from doxorubicin-induced stress**

Maciej Pudełek, Damian Ryszawy^†^, Katarzyna Piwowarczyk, Sławomir Lasota, Zbigniew Madeja, Sylwia Kędracka-Krok and Jarosław Czyż


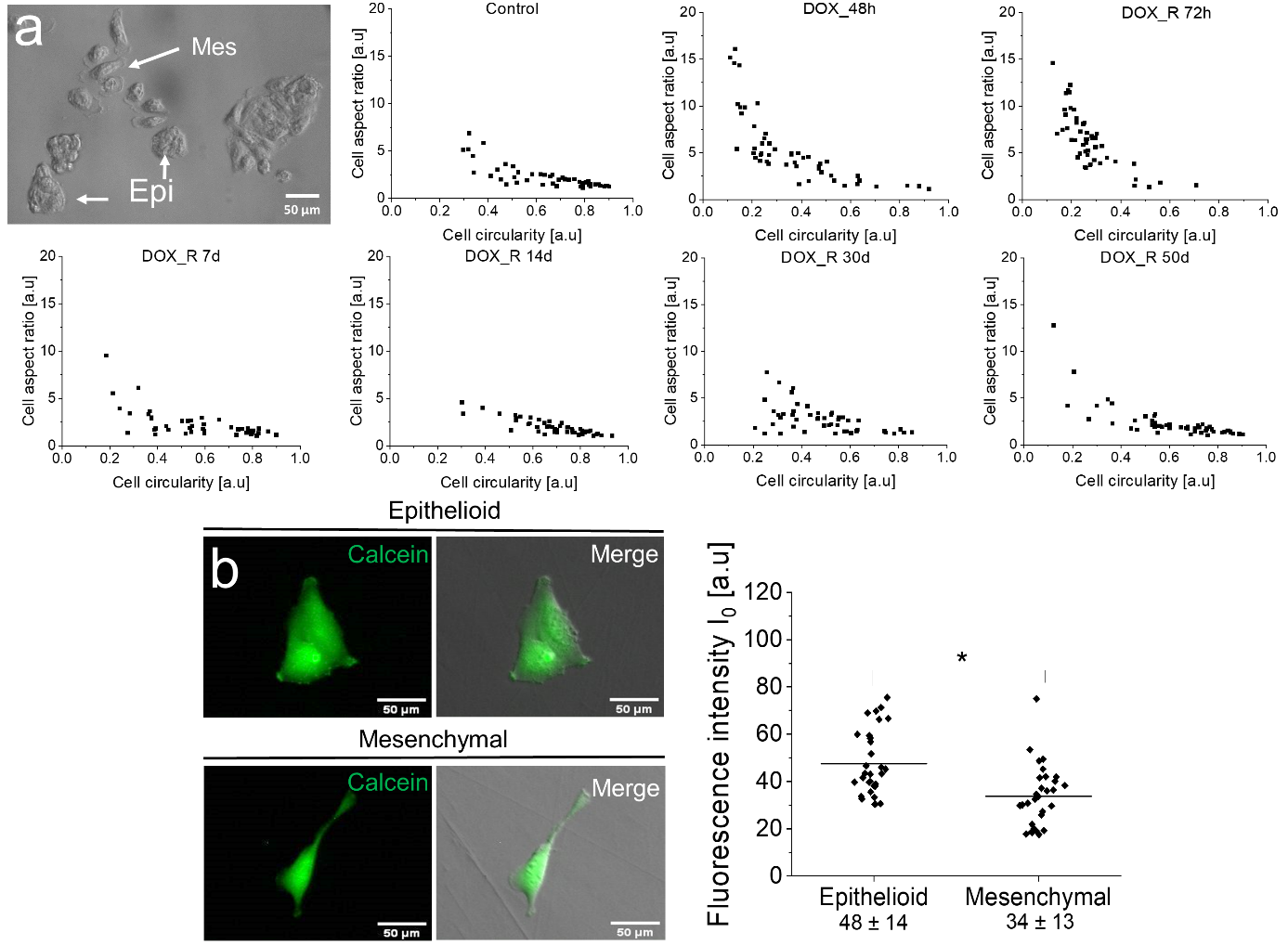


**Figure S1. Heterogeneity of T98G populations. (a)** Morphologic diversity of T98G cells undergone a pulse DOX treatment (1 μM, 48 hours), illustrated by their “elongation” (aspect ratio) and “circularity” estimated with ImageJ software. **(b)** Relationship between morphology and drug-resistance of T98G cells estimated with calcein-efflux assay. Representative images of calcein-loaded epithelioid and mesenchymal T98G cells. Data representative for ≥30 single cells and 3 independent biological replicates (N=3). Statistical significance was calculated with t-student test, *p<0.05 vs. control. Scale bars = 50 µm. **Note the correlation between “mesenchymal” morphology and drug-efflux inT98G cells, followed by a transient increase of the fraction of non-circular/elongated cells after a pulse DOX treatment.**

***
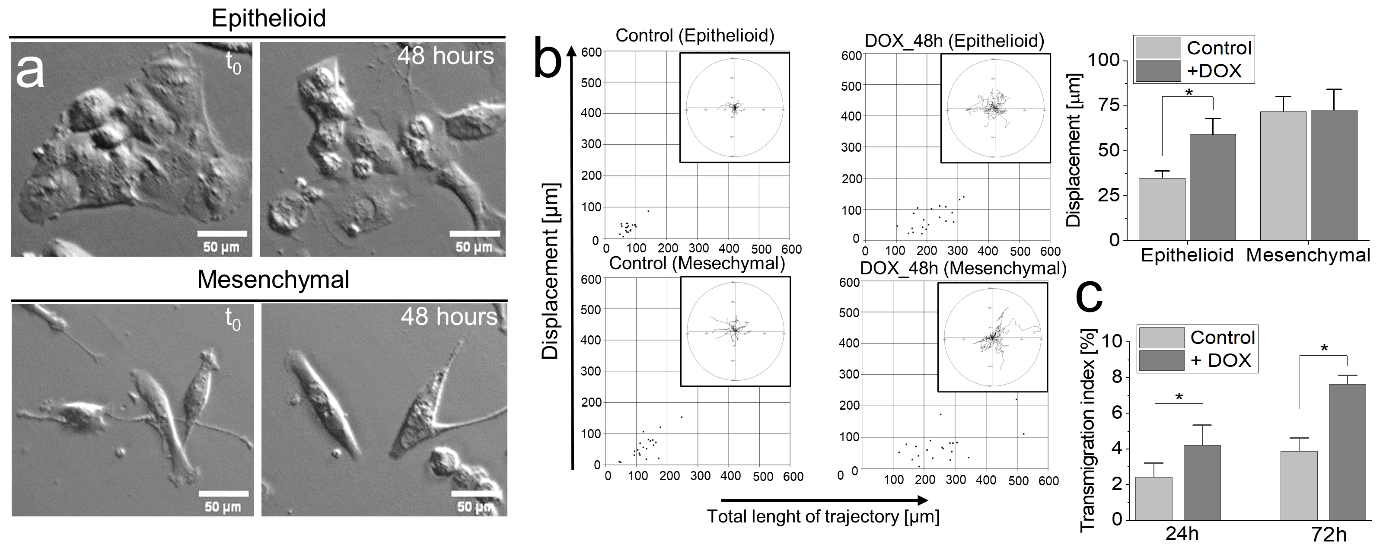
***

**Figure S2. Reactivity of “epithelioid” and “mesenchymal” T98G cells to doxorubicin. (a)** Morphology of epithelioid and mesenchymal T98G cells in control conditions (t_0_) and after 1 μM DOX-treatment (48 hours). **(b, c)** T98G cells were incubated in the presence of doxorubicin (1 µM) for 48 hours and their motility **(b)** and invasiveness **(c)** was estimated with the NIC microscopy-assisted time-lapse videomicroscopy and Transwell® assay, respectively. Circular diagrams, dot-plots and bar graphs show single cell trajectories, movement parameters (total length of cell trajectory (i.e. the distance) and displacement) at the single cell and population level. Transmigration index was calculated for entire cell populations. Data representative for ≥30 single cells and/or 3 independent biological replicates (N=3). Statistical significance was calculated with the non-parametric Mann-Whitney (b) or t-student test (c), *p<0.05 vs. control. Scale bars = 50 µm. **Note differential DOX-reactivity of “epithelioid” and “mesenchymal” T09G cells.**

**
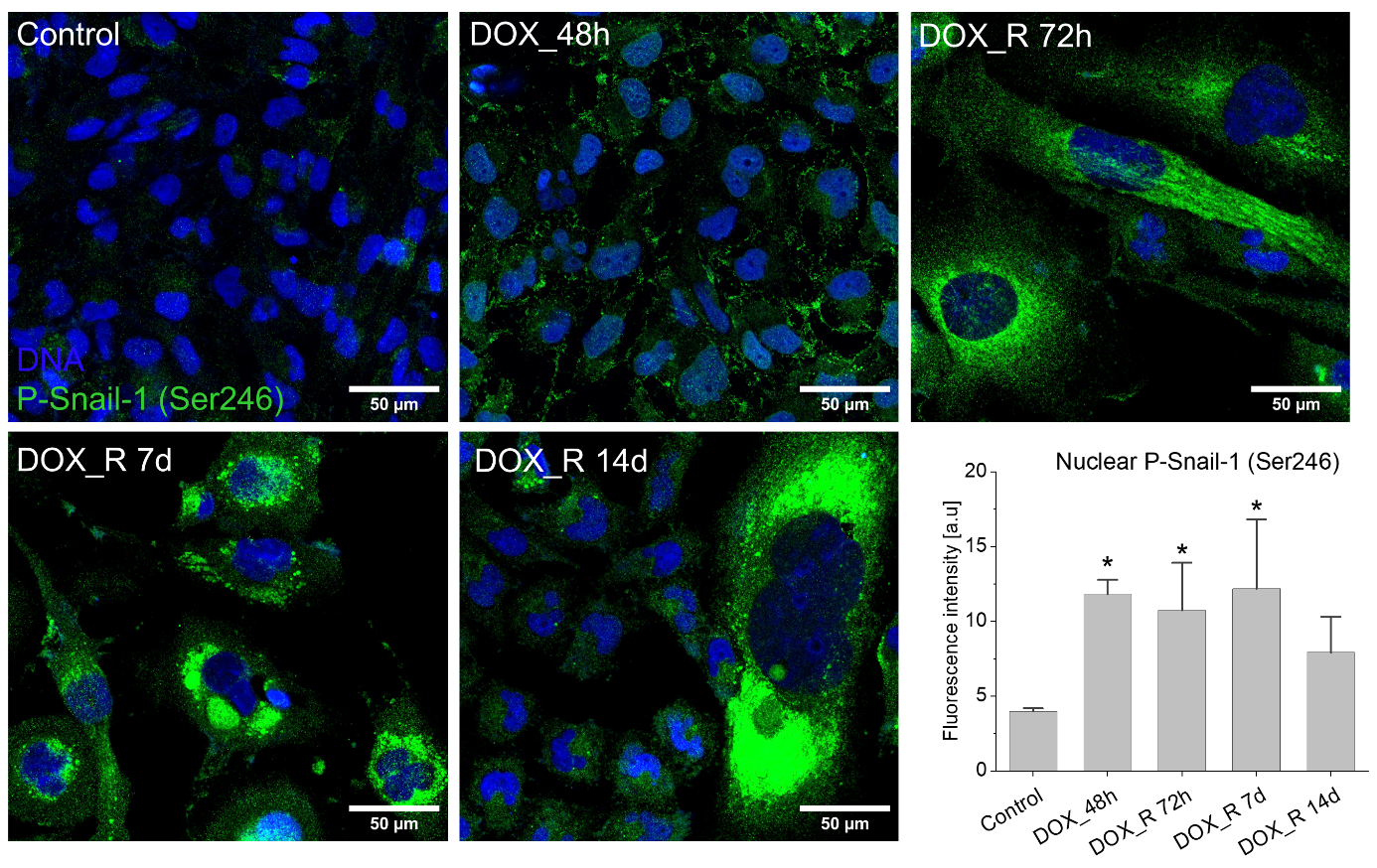
**

**Figure S3. DOX-induced EMT in T98G populations.** Fluorimetric quantification of nuclear p-Snail-1(Ser246)-specific staining. Data representative for 3 independent biological replicates (N=3). Statistical significance was calculated with ANOVA followed by Tukey post-hoc test, *p<0.05 vs. control. Scale bars = 50 µm. **Note increased pSnail-1 levels and nuclear accumulation following pulse DOX treatment of T98G cells.**


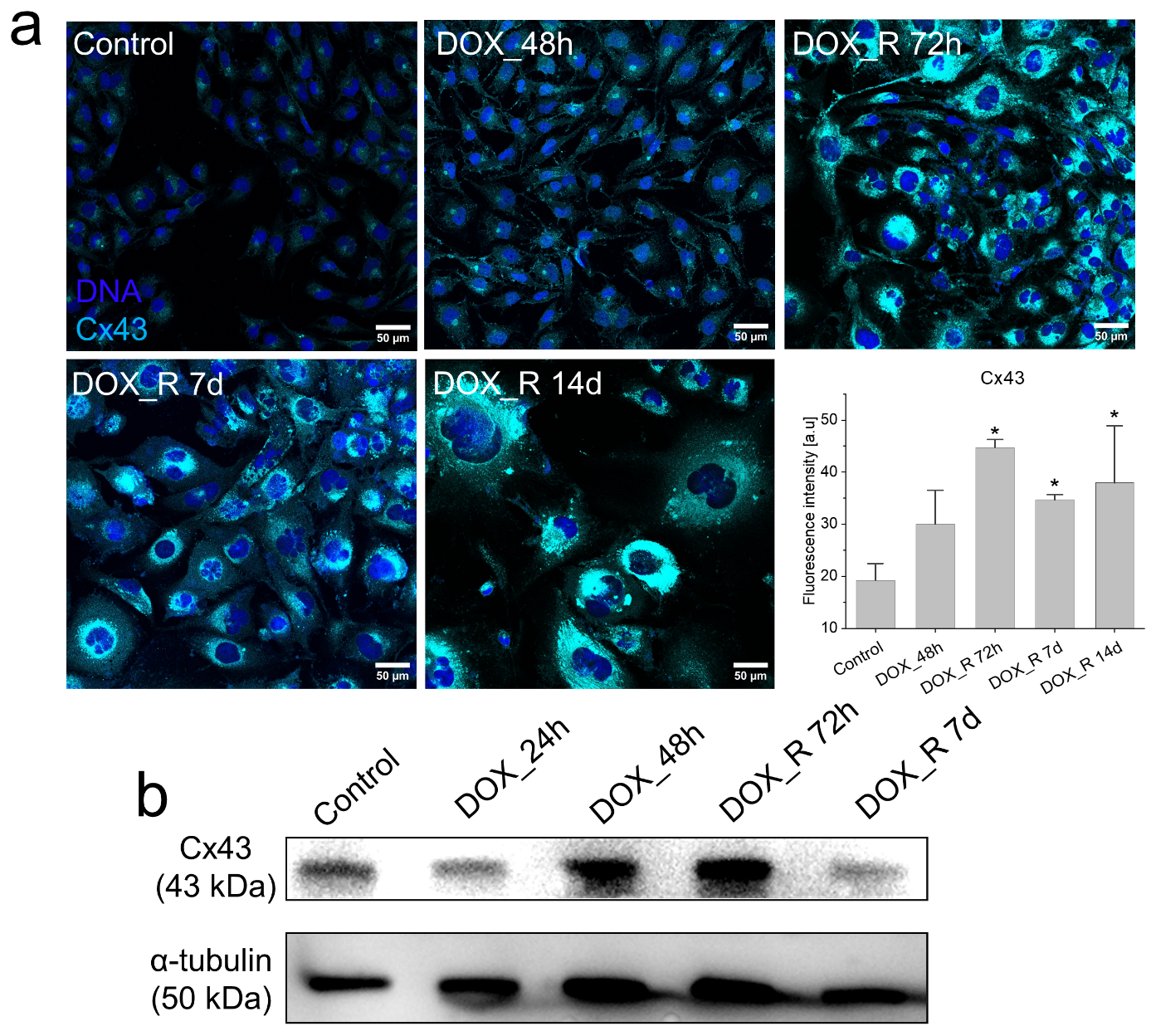


**Figure S4. DOX-induced EMT in T98G populations.** Fluorimetric quantification and immunoblot visualization of Cx43 in T98G cells in DOX presence and after its removal. Data representative for 3 independent biological replicates (N=3). Statistical significance was calculated with ANOVA followed by Tukey post-hoc test, *p<0.05 vs. control. Scale bars = 50 µm. **Note the transient up-regulation of Cx43 following a pulse DOX treatment of T98G cells.**

**
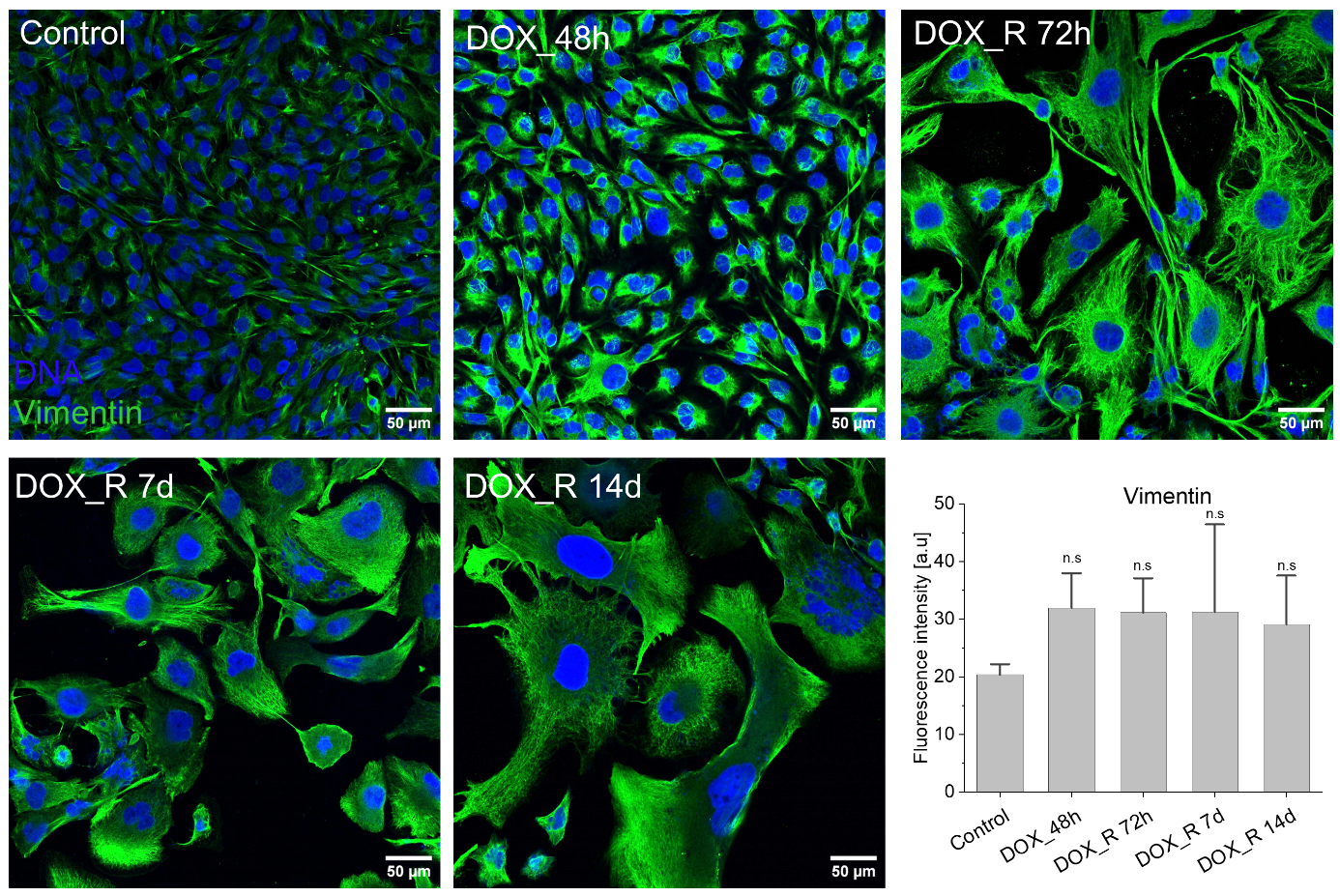
**

**Figure S5. DOX-induced EMT in T98G populations.** Fluorimetric quantification of vimentin levels in T98G cells in DOX presence and after its removal. Data representative for 3 independent biological replicates (N=3). Statistical significance was analyzed with ANOVA followed by Tukey post-hoc test vs. control. Scale bars = 50 µm. **Note increased levels of vimentin following a pulse DOX treatment of T98G cells.**

**
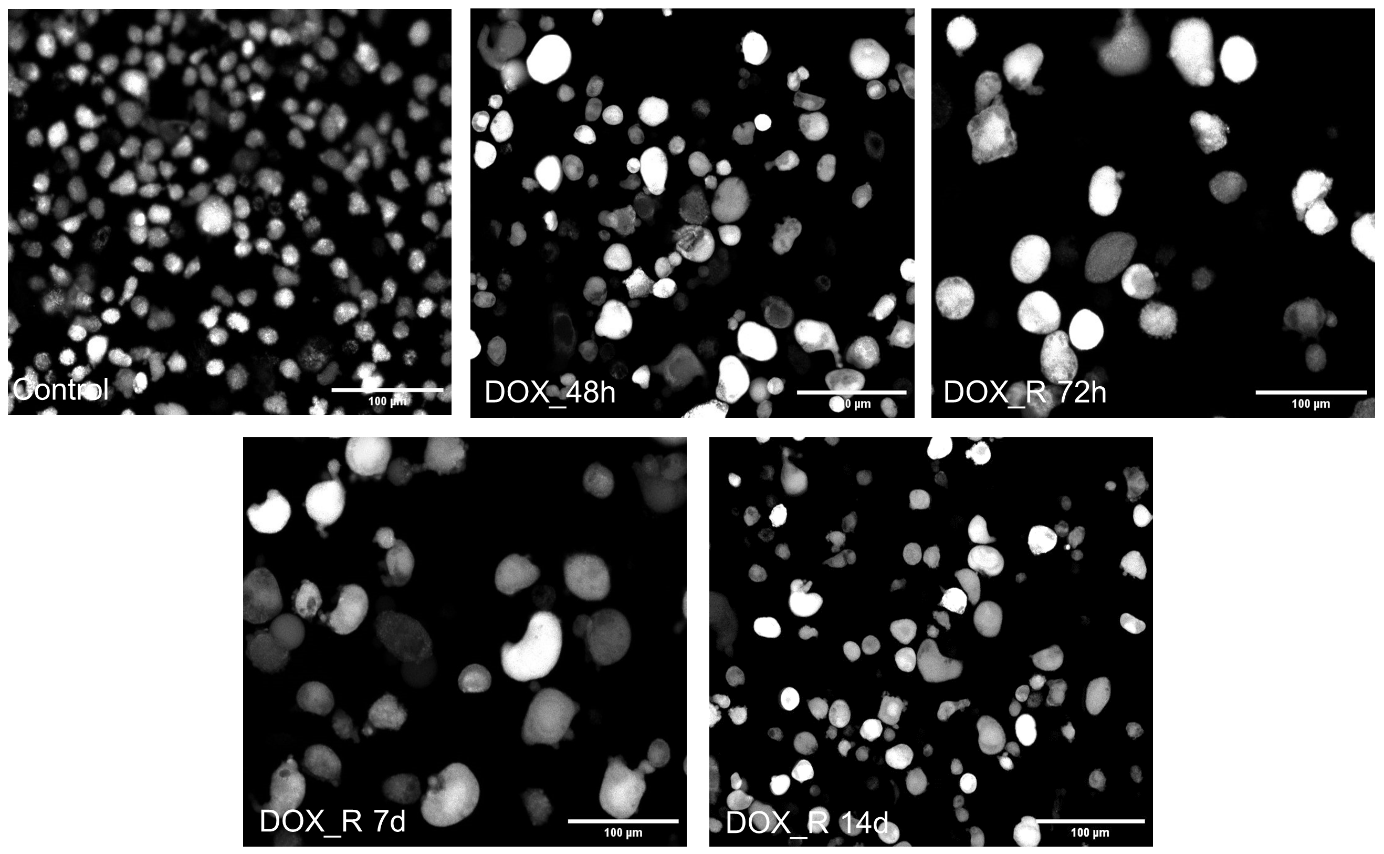
**

**Figure S6. Hypertrophy of T98G cells following pulse DOX treatment.** Cells were suspended at the indicated time-points after DOX removal, loaded with 1 μM calcein AM and visualized with fluorescence microscopy. Scale bars = 100 µm. **Note an increase of cell volumes of T98G cells exposed to pulse DOX treatment.**


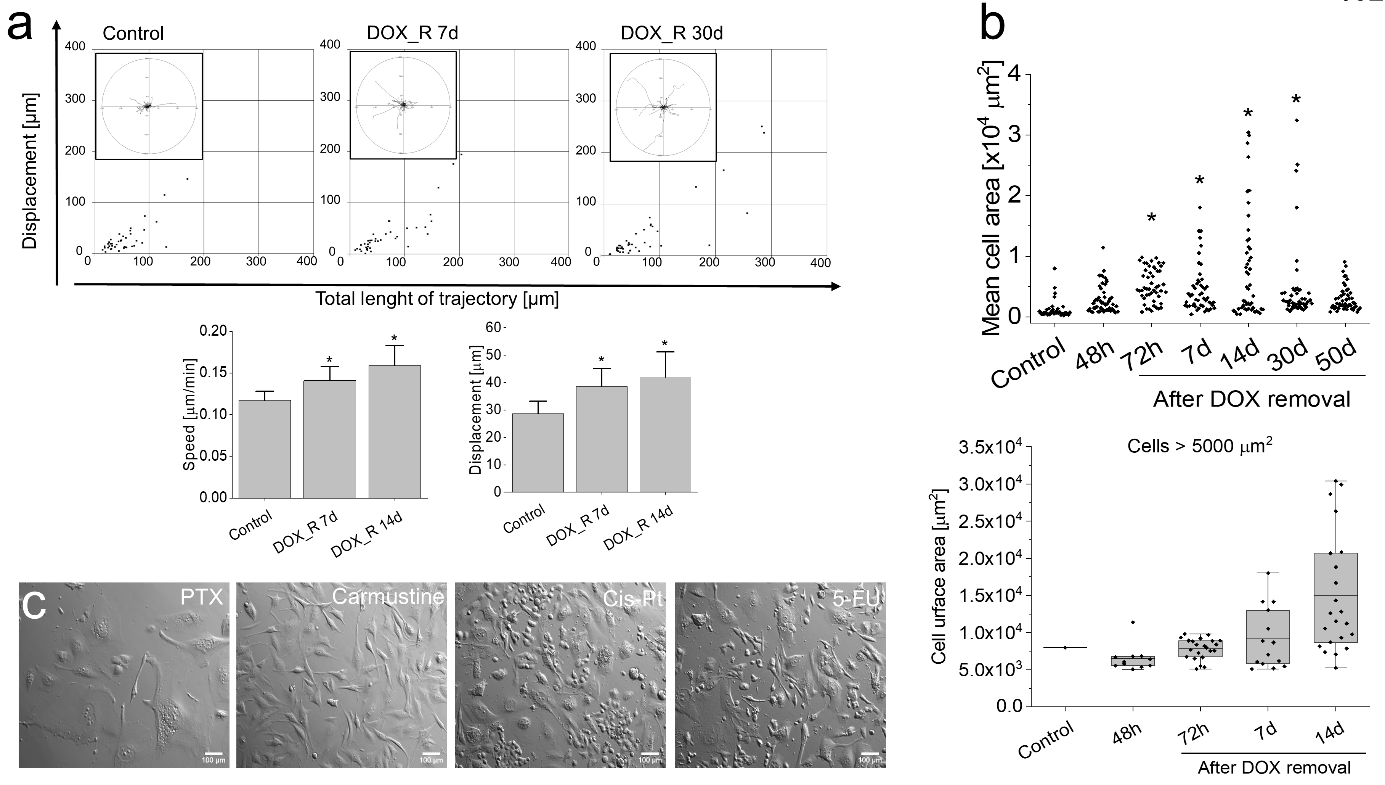


**Figure S7. Long-term DOX effects in T98G populations. (a)** T98G cells were incubated in the presence of doxorubicin (1 µM) for 48 hours and the motility of PGCs was estimated with time-lapse videomicroscopy at the indicated time-points. Circular diagrams, dot-plots and bar graphs show single cell trajectories, movement parameters (distance and displacement) at the single cell and population level. **(b)** Projection areas of T98G cells following a pulse DOX treatment, estimated with ImageJ-assisted morphometry. **(c)** Long-term effect of the pulse PTX, carmustin, cis-platin and 5-fluoruracyl treatment on the morphology of T98G cells. Data representative for ≥30 single cells and/or 3 independent biological replicates (N=3). Statistical significance was calculated with the non-parametric Mann-Whitney test (a) and ANOVA followed by Tukey post-hoc test (b), *p<0.05 vs. control. Scale bars = 100 µm. **Note the relatively high motile activity of PGCs and their dynamics under chemotherapeutic stress.**


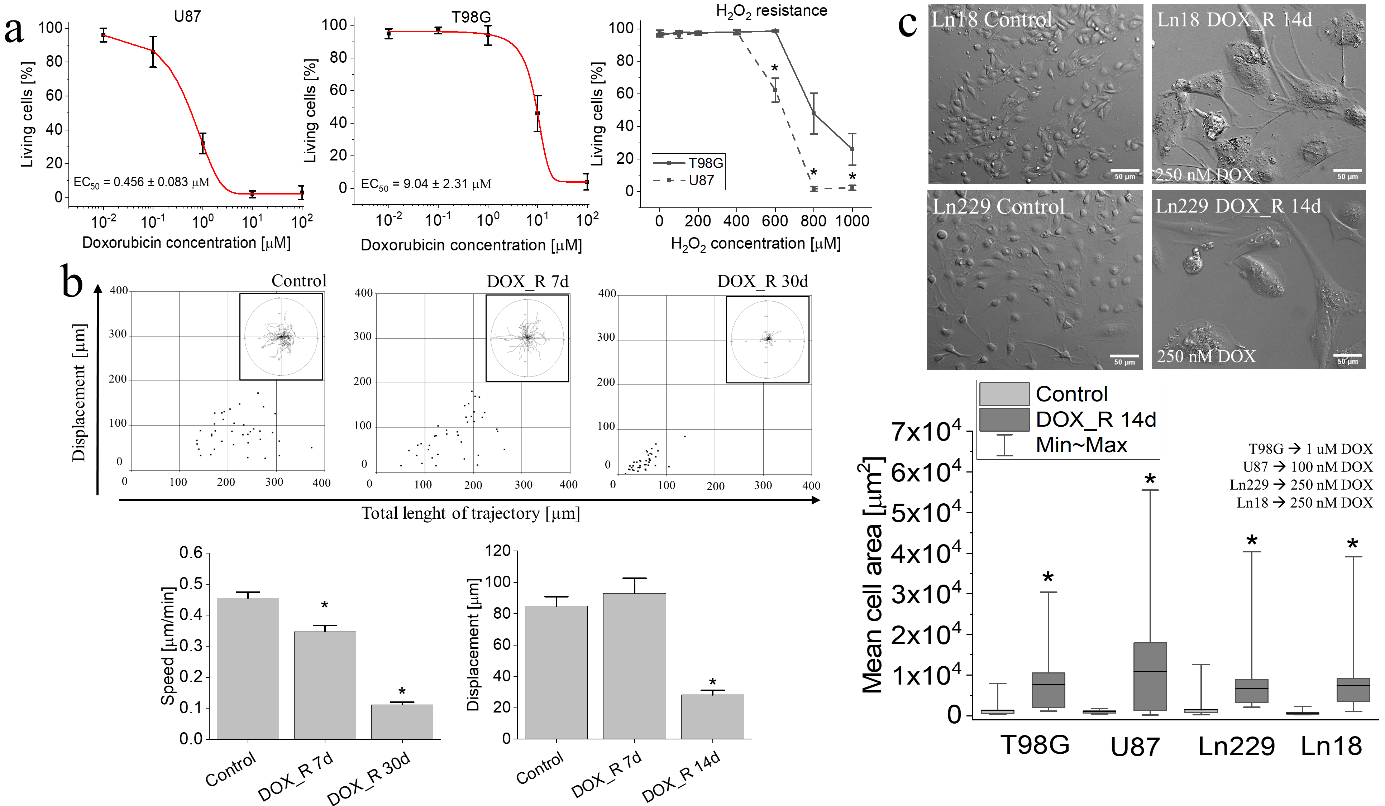


**Figure S8. Cytotoxic/cytostatic effects of DOX in GBM populations. (a)** T98G and U87 cells were incubated in the presence of doxorubicin (10 nM-100 µM; DOX). Their viability (EC_50_ values; left) and resistance to oxidative stress (H_2_O_2_; right) was estimated 48 hours after DOX administration with trypan blue assay. **(b)** Motility of U87 cells undergone a pulse (1 µM, 48 hours) DOX treatment estimated with time-lapse videomicroscopy. Circular diagrams, dot-plots and bar graphs show single cell trajectories, movement parameters (distance and displacement) at the single cells and population level. **(c)** Long-term effect of the pulse DOX-treatment on the morphology of T98G (1 µM), U87 (100 nM), Ln229 and Ln18 cells (250 nM) estimated 14 days after drug removal. Statistical significance was calculated with t-student (a), Mann-Whitney (b) and ANOVA followed by Tukey post-hoc test (c), *p<0.05 vs. control. Data representative for ≥ 30 single cells and/or 3 independent biological replicates (N=3). Scale bars = 50 µm. **Note the high sensitivity of U87 cells to DOX-induced stress and the DOX dose-dependent hypertrophic response of GBM cells.** Due to the lower DOX resistance of U87-MG cells (IC50 = 0.456±0.083 µM vs. 9.04±2.31 µM for T98G cells), 1 μM DOX strongly interfered with their viability and motility **(a,b)**, whereas giant cell formation in U87-MG populations was observed in the presence of 100 nM DOX **(c)**. In Ln229 and Ln18 populations, such cells were generated after the administration of 250 nM DOX **(c)**.


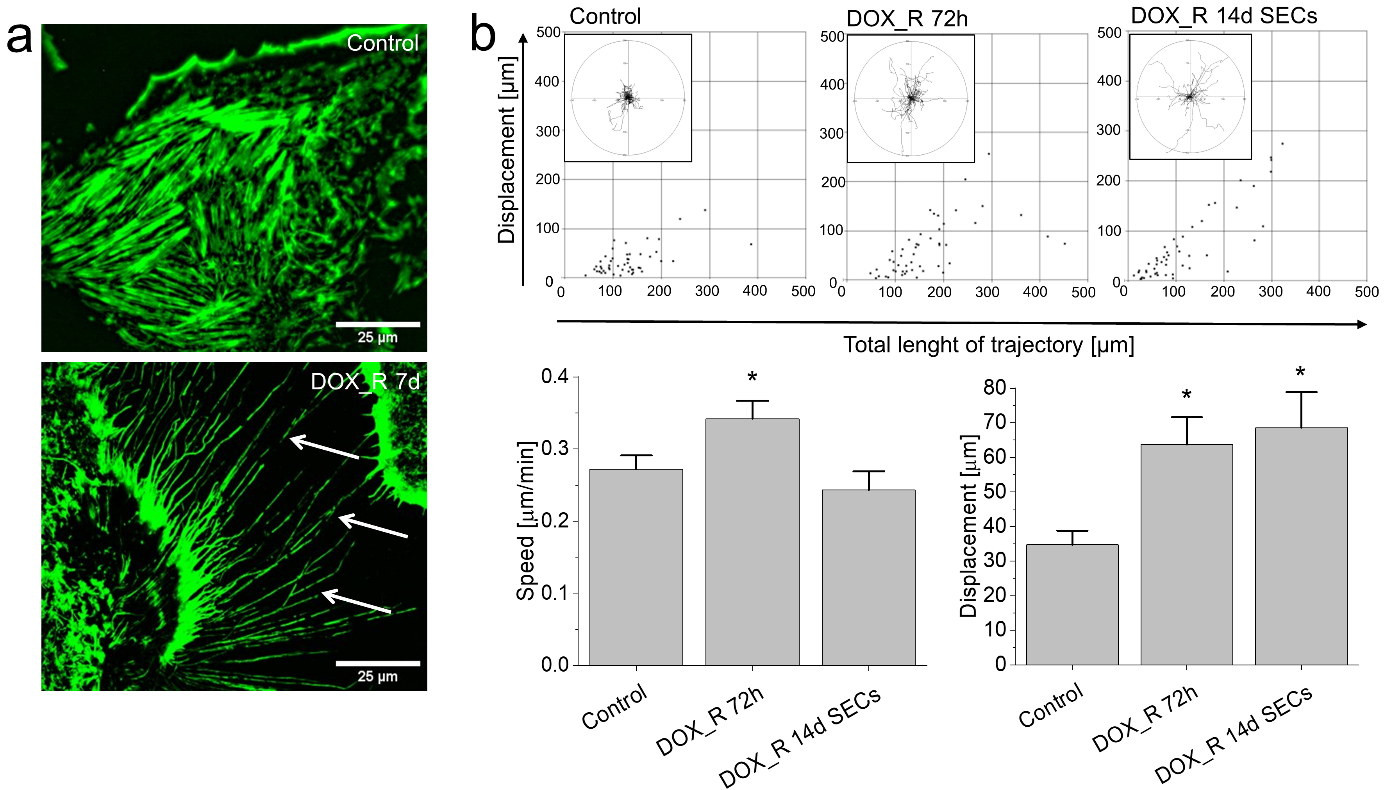


**Figure S9. Cooperative microevolution in DOX-treated T98G populations. (a)** Microvilli at the perimeters of DOX-induced PGCs (2+14^th^ day) visualised with CLSM. **(b)** Motility of clustered SECs. Cells were incubated in the presence of doxorubicin (1 µM) for 48 hours. The motility of the clustered SECs was estimated with time-lapse videomicroscopy at the 14^th^ day after DOX removal and compared with the motility of their counterparts cultured in control conditions and 3 days after DOX removal Circular diagrams, dot-plots and bar graphs show single cell trajectories, movement parameters (distance and displacement) at the single cell and population level. Statistical significance was calculated with Mann-Whitney test, *p<0.05 vs. control. Scale bars = 25 µm. Data representative for ≥ 30 single cells in 3 independent biological replicates (N=3). **Note a relatively high motile activity of SECs.**


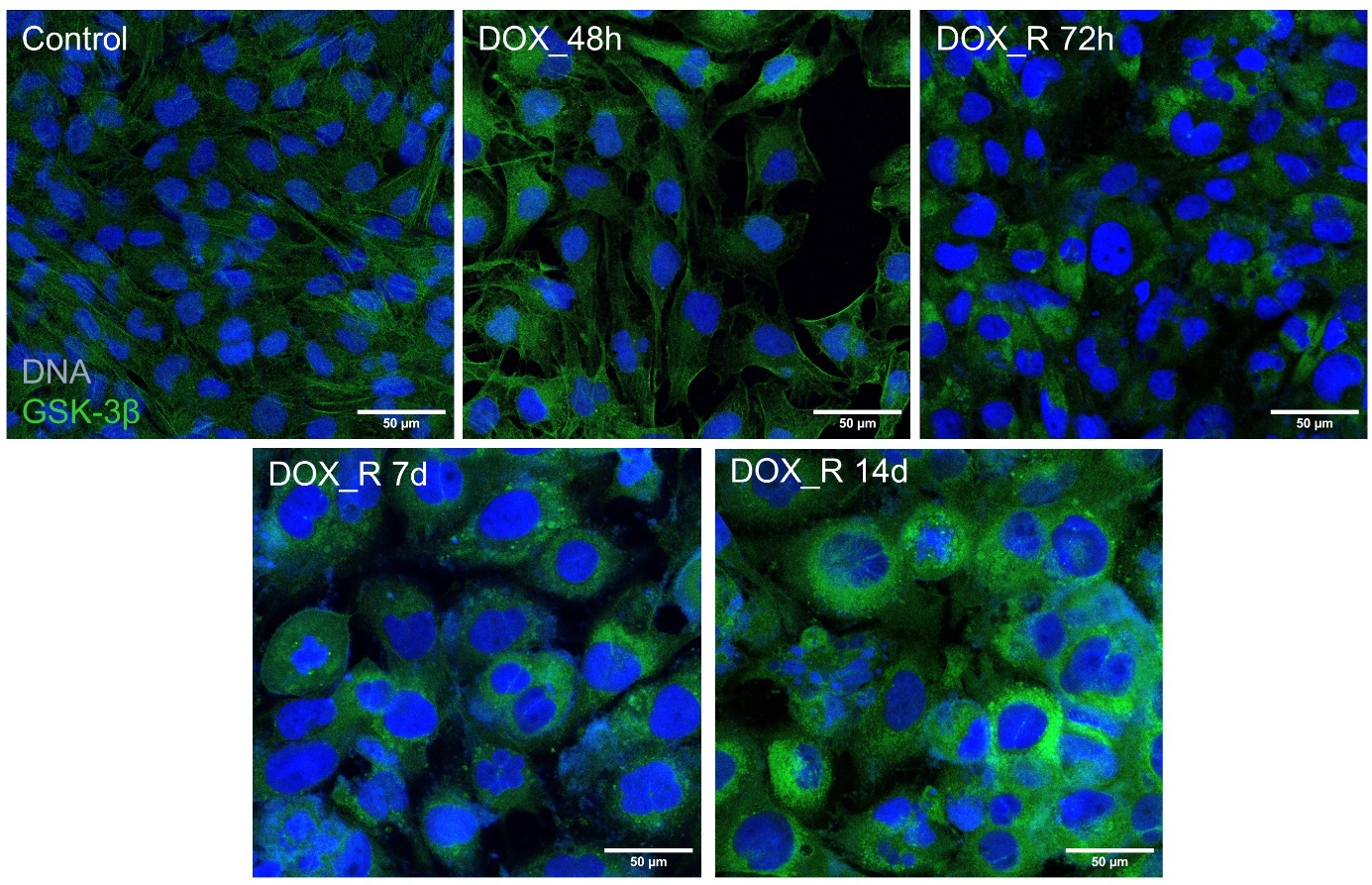


**Figure S10.** Fluorimetric quantification of GSK-3β levels in T98G cells in DOX presence and at the indicated time-points after DOX removal. Data representative for 3 independent biological replicates (N=3). Scale bars = 50 µm. **Note GSK-3β up-regulation following the pulse DOX treatment of T98G cells.**

**
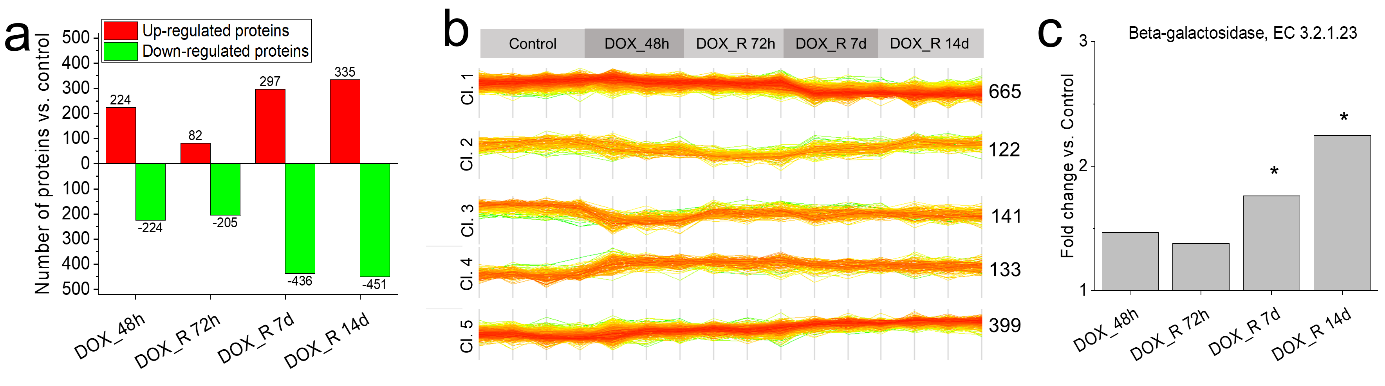
**

**Figure S11. Proteomic profile of DOX-induced PGCs. (a,b)** Large-scale proteomic shifts in T98G cells undergone a pulse DOX treatment estimated at the indicated time-points **(a)** and the clusters of proteins discerned with LC-MS/MS proteomic approach according to the dynamics of their levels. **(c)** Time-course of β-galactosidase levels in T98G cells following a pulse DOX treatment. Statistical significance was calculated with ANOVA permutation based FDR<0.05 and post hoc Tukey’s test, *p<0.05 vs. control. **Note robust DOX-induced proteomic shifts in T98G PGCs.**


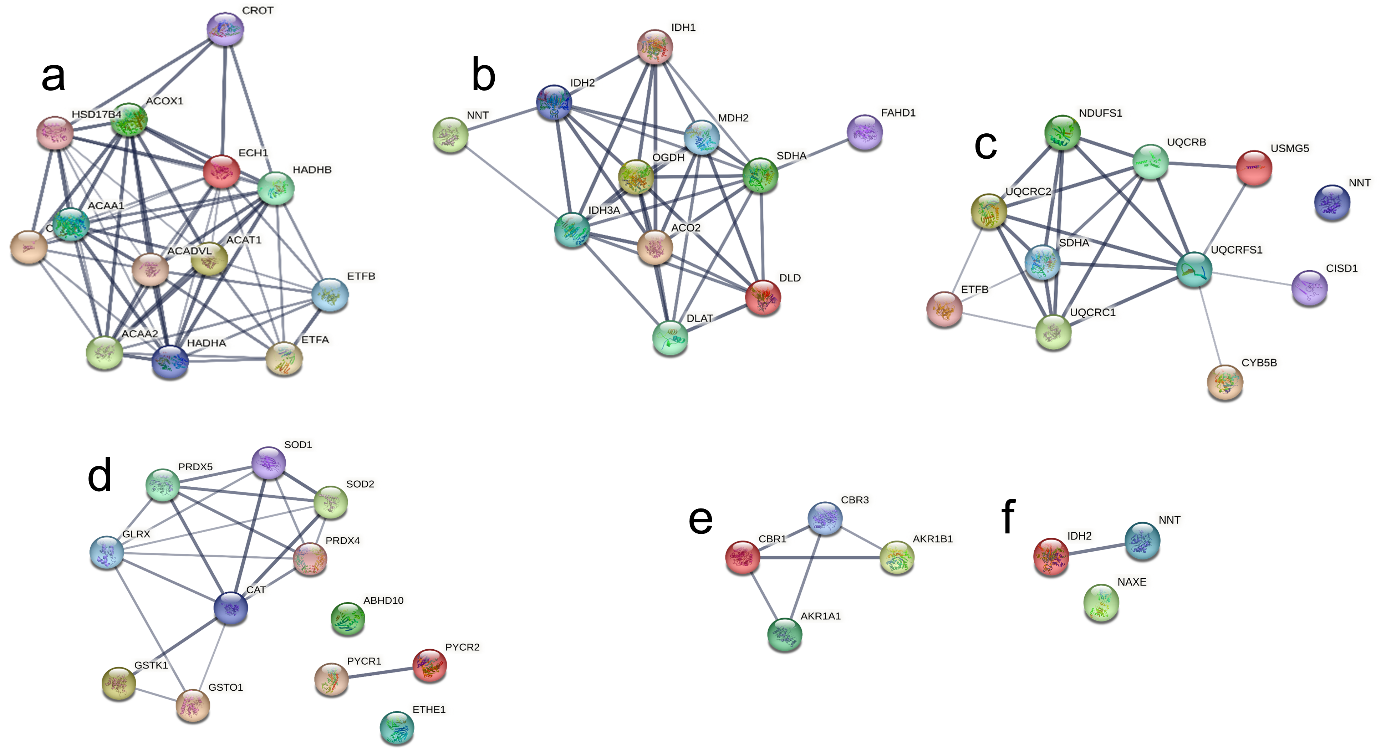


**Figure S12. STRING-generated interactomes of the clusters of up-regulated proteins** involved in the β-oxidation **(a)**, Krebs cycle **(b)**, oxidative phosphorylation **(c)**, redox homeostasis **(d**), DOX degradation **(e)** and NADPH balancing **(f)** revealed by LC-MS/MS proteomic approach in DOX-induced T98G PGCs at the 14^th^ day after DOX removal. Data representative for 3 independent biological replicates (N=3). **Note the coordinated mobilization of metabolic and self-defense systems in DOX-induced PGCs.**

**
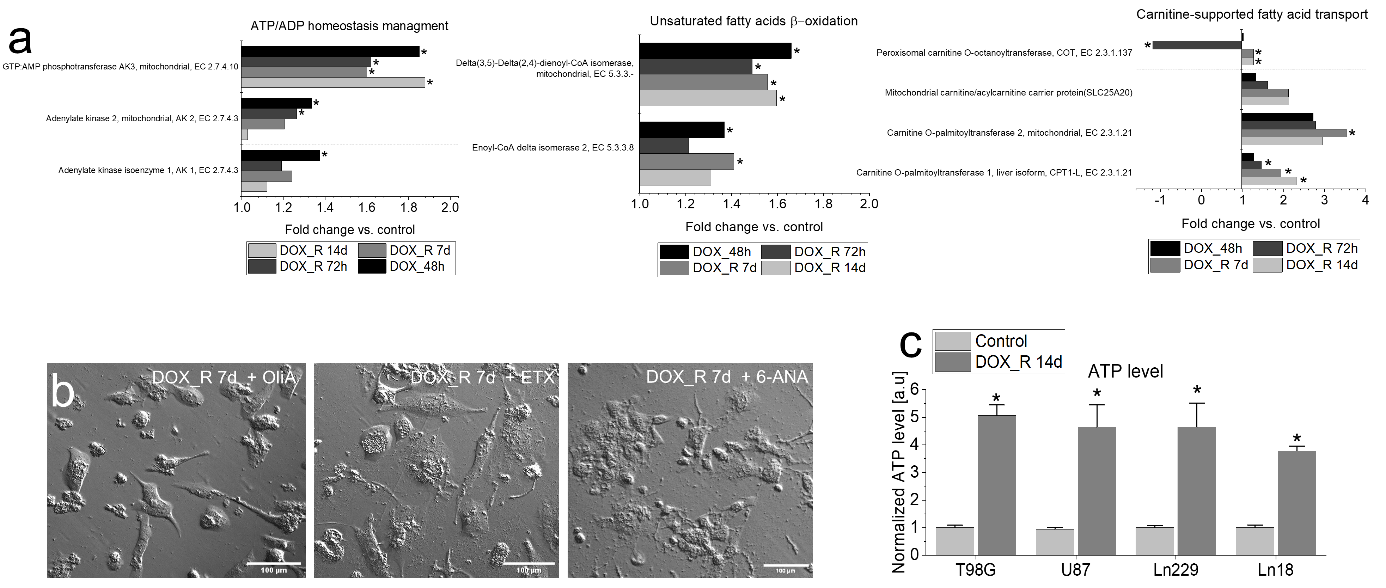
**

**Figure S13. DOX-induced metabolic reprogramming of T98G cells. (a)** Clusters of up-regulated proteins involved in the transport of carnitine, ATP/ADP homeostasis and the oxidation of unsaturated fatty acids revealed by LC-MS/MS proteomic approach. **(b)** The effect of metabolic blockers on the morphology of DOX-pretreated T98G PGCs. **(c)** ATP accumulation in U87, Ln229 and Ln18 PGCs following a pulse DOX-treatment. Data representative for 3 independent biological replicates (N=3). Statistical significance was calculated with ANOVA permutation based FDR<0.05 and post hoc Tukey’s test (a) or t-student test (c), *p<0.05 vs. control. Scale bars = 100 µm. **Note the metabolic mobilization of PGCs metabolic apparatus and the interference of OliA and ETX with the viability of T98G.**


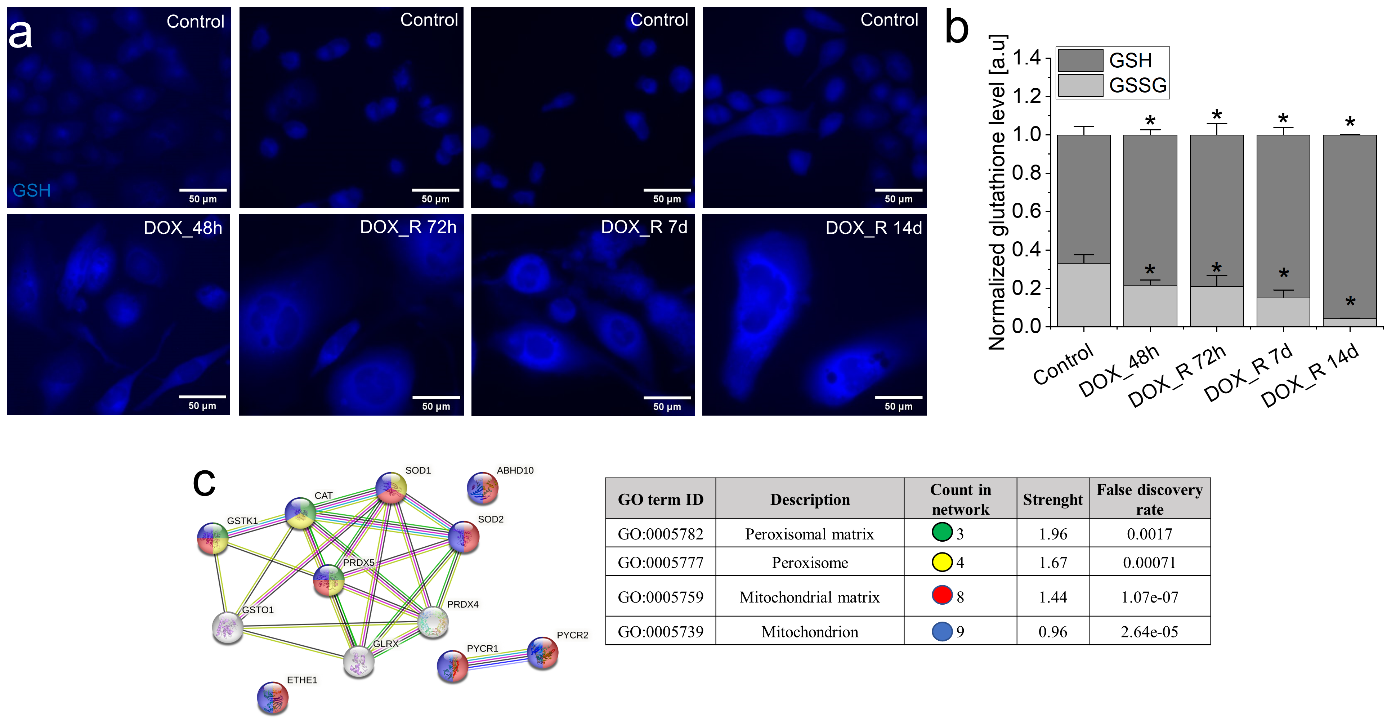


**Figure S14. Mobilization of antioxidative systems in DOX-treated T98G cells.** **(a)** GSH levels in T98G PGCs estimated with the ThiolTracker^TM^ Violet assay at the indicated time-points. **(b)** GSH and GSSG levels in pulse DOX treated T98G cells with the Glutathione GSH/GSSG Assay Kit. **(c)** The cluster of ROS managing proteins revealed by LC-MS/MS proteomic analyses along with their STRING-generated interactome. Data representative for 3 independent biological replicates (N=3). Scale bars = 50 µm. **Note the mobilization of ROS scavenger systems in PGCs.**


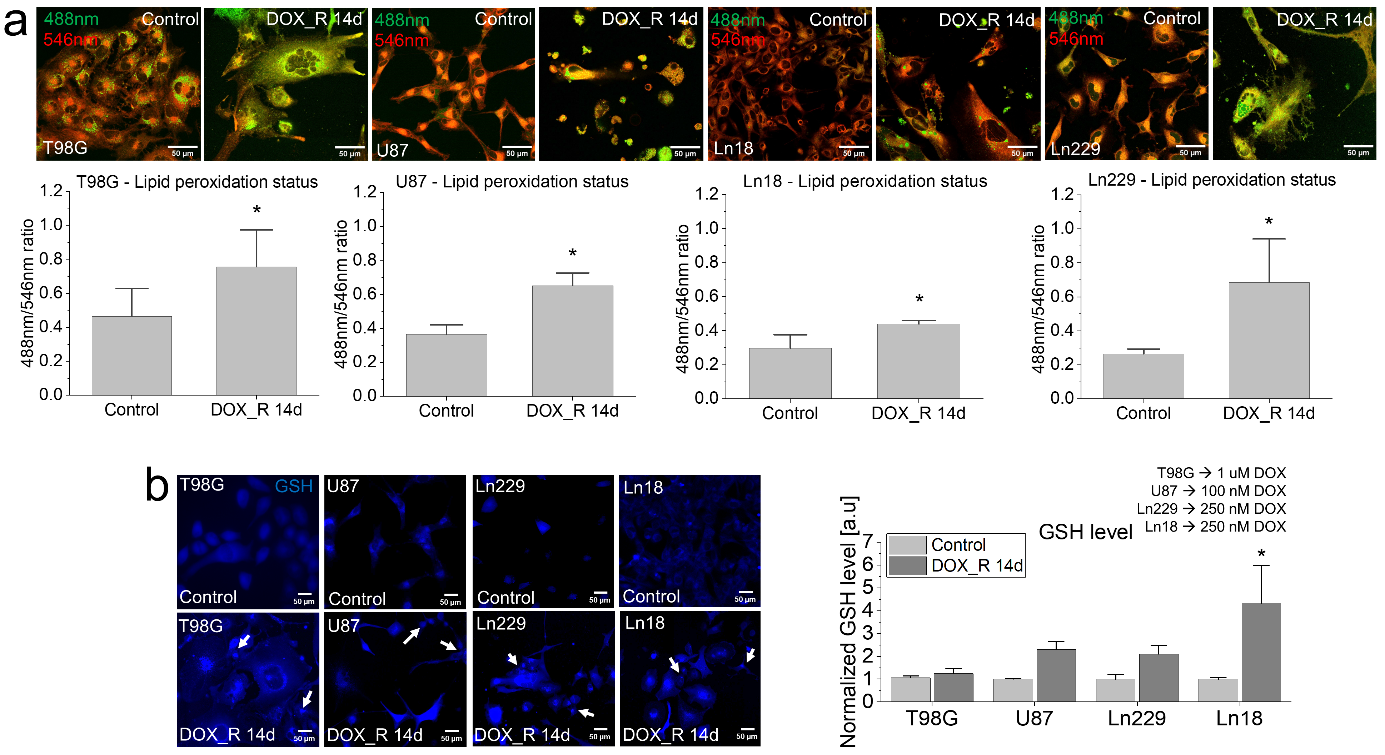


**Figure S15. Long-term ROS management in GBM cells. (a)** Long-term DOX effects on the lipid peroxidation in U87 (100 nM DOX), Ln18 and Ln229 cells (250 nM DOX) estimated with ImageiT^TM^ Lipid Peroxidation kit. **(b)** Intracellular GSH content in DOX-treated U87, Ln229 and Ln18 cells estimated with the fluorescence microscopy-assisted (ThiolTracker^TM^ Violet assay) 14 days after DOX removal. Data representative for ≥30 single cells in 3 independent biological replicates (N=3). Statistical significance was calculated with t-student (a) and ANOVA and Tukey’s post hoc test (b), *p<0.05 vs. control. Scale bars = 50 µm. **Note the mobilisation of self-defence systems in U87, Ln229 and Ln18 PGCs, accompanied by the formation of SEC clusters (arrows).**


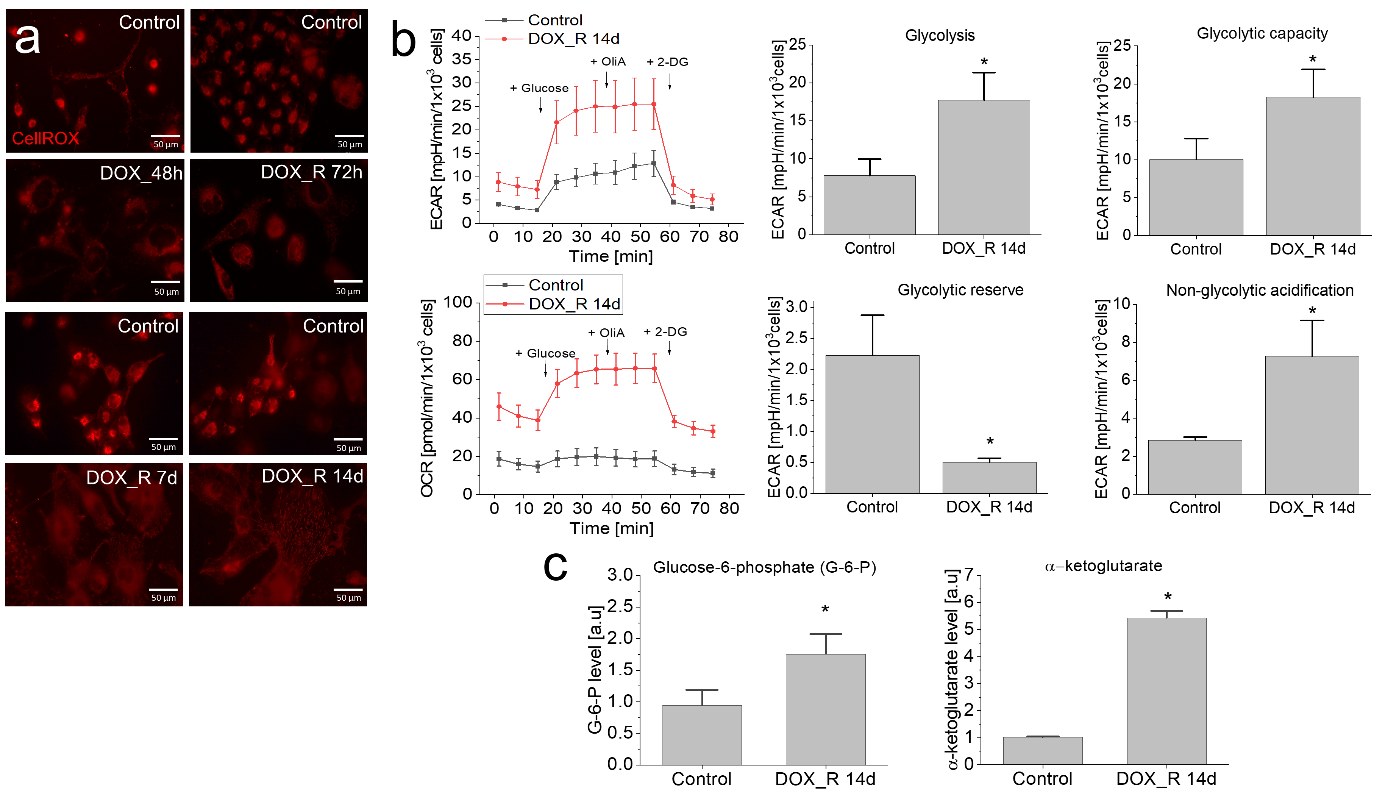


**Figure S16. Metabolic profile of T98G PGCs. (a)** Mitochondrial ROS levels estimated with fluorescence microscopy-assisted CellROX DeepRed dye assay. **(b)** Seahorse XFp analyses of the long-term DOX effects on the glycolytic activity of T98G cells. **(c)** Glucose-6-phosphate and α-ketoglutarate levels in DOX-induced PGCs. Statistical significance was calculated with t-student test, *p<0.05 vs. control. Data representative for 3 independent biological replicates (N=3). Scale bars = 50 µm. **Note** **the negligible mitochondrial ROS in PGCs accompanied by the activation of glycolysis to the limits of PGC glycolytic reserve.**


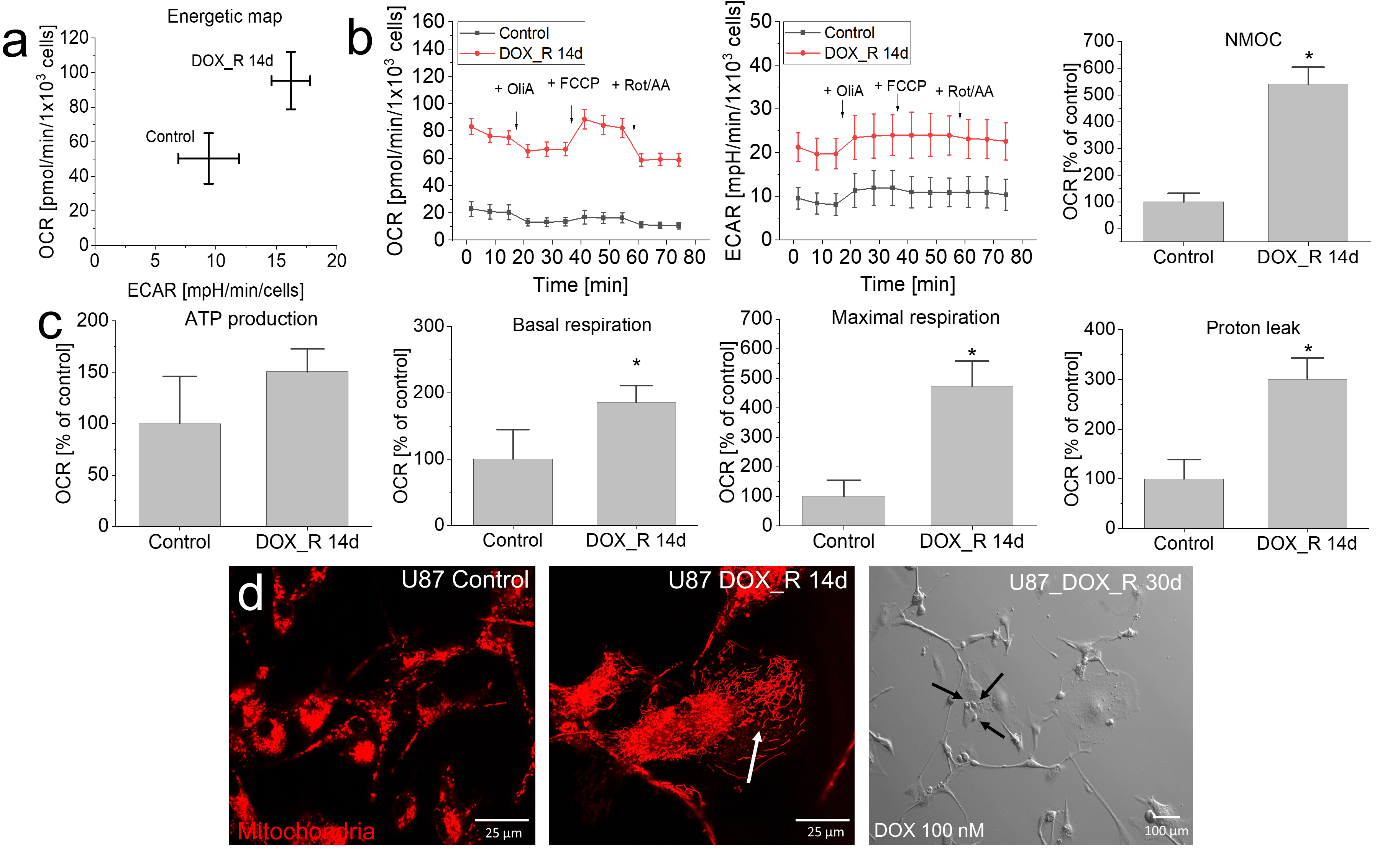


**Figure S17. DOX-induced metabolic reprogramming of U87 cells and its long-term consequences.** **(a-c)** Seahorse XFp analyses of the long-term effect of pulse DOX treatment (100 nM, 14 days after DOX removal) on the oxygen consumption rate (OCR), extracellular acidification rate (ECAR, a) and the parameters of (non)mitochondrial ATP production in U87 cell (b-c). **(d)** Architecture of mitochondria in U87 PGCs (left) and the cohabitation of PGCs and SECs in U87 populations 14 days after DOX removal (right). Statistical significance of the differences was calculated with t-student test, *p<0.05 vs. control. Data representative for 3 independent biological replicates (N=3). Scale bars = 25 and 100 µm. **Note the DOX-induced mobilization of energy production in U87 cells, accompanied by the mitochondrial fusion and the formation of expanding clusters of non-hypertrophic cells (arrows).**


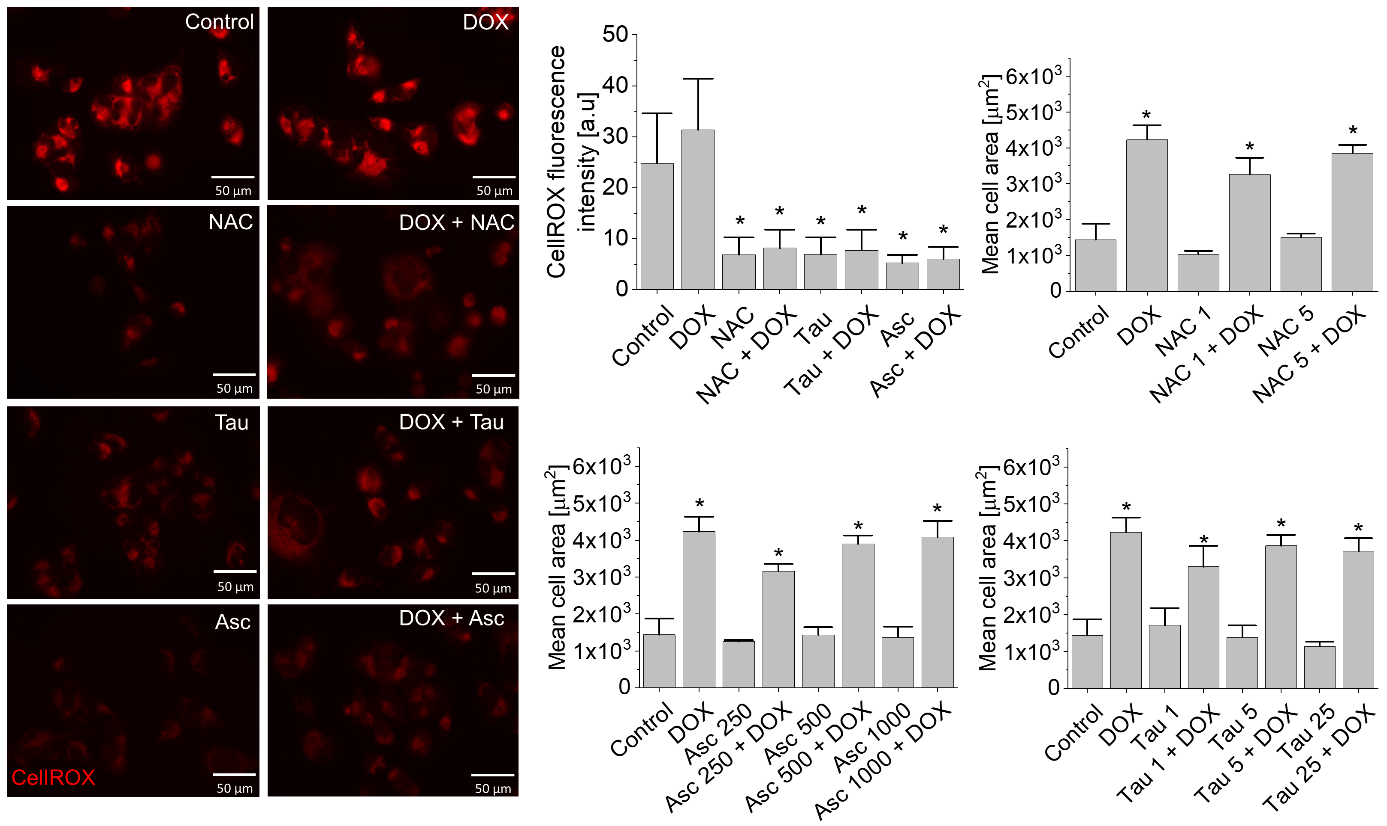


**Figure S18.** Effect of N-acetyl-L-cysteine (NAC), Taurine (Tau) and Sodium Ascorbate (Asc) on the ROS levels and the efficiency of PGC generation following the pulse DOX-treatment. Images show the cells treated with DOX (1 µM) and antioxidants (N-Acetyl-L-cystine (NAC); 5 mM; Taurine (Tau); 5 mM; Sodium ascorbate (Asc); 500 µM) for 48 hours. Statistical significance of the differences was calculated with t-student test, *p<0.05 vs. control. Data representative for 3 independent biological replicates (N=3). Scale bars = 50 µm. **Note the lack of NAC/Tau/ASC interference with DOX-induced PGC formation in T98G populations.**

***
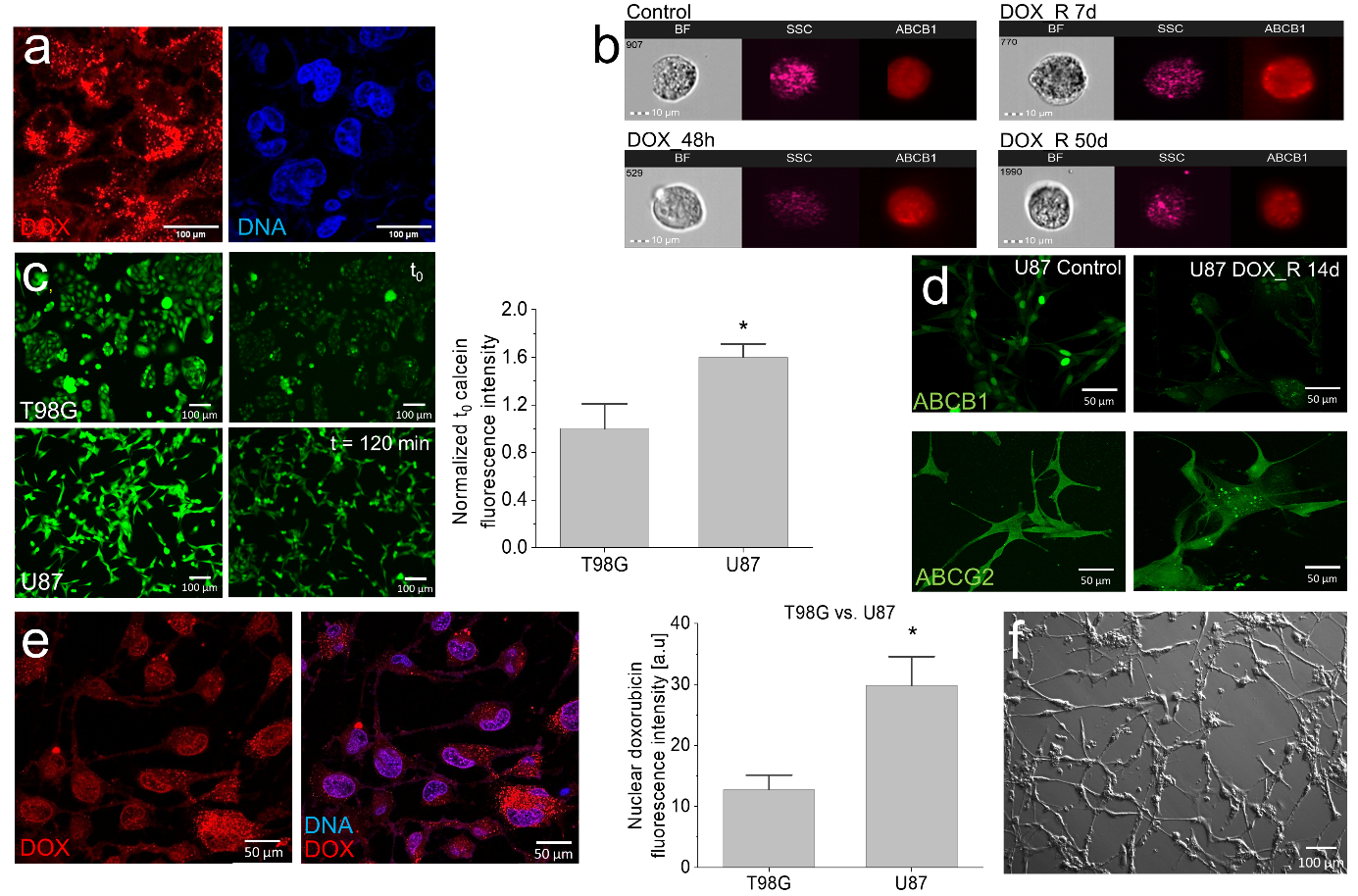
***

**Figure S19. Drug-retention and the activity of ABC transporters in T98G and U87 cells. (a, b)** Representative photomicrographs (a) and ImageStream images (b) of intracellular DOX localization in T98G cells. **(c)** Drug-efflux efficiency in U87 and T98G populations estimated with fluorimetry-assisted calcein efflux assay. **(d)** Intracellular localization of ABCB1 and ABCG2 in U87 cells in control conditions and following the DOX treatment (14 days after DOX removal; cf. Fig. 7). **(e,f)** Nuclear DOX accumulation following a pulse DOX-treatment of U87 cells (e) and its correlation with DOX cytotoxicity (f). Statistical significance of the differences was calculated with t-student test, *p<0.05 vs. control. Data representative for 3 independent biological replicates (N=3). Bars represent SD values. Scale bars = 100 µm (a,c,f) and 50 µm (e,d). **Note the correlation between the efficiency of drug transporters, nuclear DOX accumulation and DOX sensitivity of T98G and U87 populations.**
