# Supplementary material for: Metabolic reprogramming of poly(morpho)nuclear giant cells determines glioblastoma recovery from doxorubicin-induced stress
Source: J Transl Med. 2024 Aug 12;22:757. doi: 10.1186/s12967-024-05541-9 (PMC11318163; doi:10.1186/s12967-024-05541-9)
Supplement: Supplementary file 2 — Supplementary Material 2 [file 12967_2024_5541_MOESM2_ESM.pdf]

Appendix 1. Proteomic data

β-oxidation involved proteins							DOX_48h vs. Control		DOX_R 72h vs. Control		DOX_R 7d vs. Control		DOX_R 14d vs. Control	
**ID**	**Protein name**	**Gene Name**	**UniProt ID**	**Unique peptides**	**p-value**	**ANOVA q-value**	**Significant**	**Fold change**	**Significant**	**Fold change**	**Significant**	**Fold change**	**Significant**	**Fold change**
**Q15067**	Peroxisomal acyl-coenzyme A oxidase 1, AOX, EC 1.3.3.6	ACOX1	ACOX1_HUMAN	17	2,196913001	0,006292035	No	1,19	No	1,17	Yes	1,46	Yes	1,26
**Q99714**	3-hydroxyacyl-CoA dehydrogenase type-2, EC 1.1.1.35	HSD17B10	HCD2_HUMAN	15	1,207282792	0,046970242	No	1,06	No	1,06	No	1,26	No	1,17
**P11310**	Medium-chain specific acyl-CoA dehydrogenase, mitochondrial, MCAD, EC 1.3.8.7	ACADM	ACADM_HUMAN	15	1,566422636	0,021621446	No	1,24	No	1,36	Yes	1,59	No	1,30
**P42765**	3-ketoacyl-CoA thiolase, mitochondrial, EC 2.3.1.16	ACAA2	THIM_HUMAN	19	5,429908615	0,000457143	Yes	1,59	Yes	1,99	Yes	2,15	Yes	2,24
**P33121**	Long-chain-fatty-acid--CoA ligase 1, EC 6.2.1.3	ACSL1	ACSL1_HUMAN	16	1,716252291	0,015808028	No	1,42	No	1,30	Yes	1,70	Yes	1,71
**P42765**	3-ketoacyl-CoA thiolase, mitochondrial, EC 2.3.1.16	ACAA2	THIM_HUMAN	19	5,429908615	0,000457143	Yes	1,59	Yes	1,99	Yes	2,15	Yes	2,24
**Q96CM8**	Medium-chain acyl-CoA ligase ACSF2, mitochondrial, EC 6.2.1.2	ACSF2	ACSF2_HUMAN	14	2,802967416	0,002472727	Yes	3,62	Yes	2,67	Yes	2,68	Yes	2,80
**P49748**	Very long-chain specific acyl-CoA dehydrogenase, mitochondrial, VLCAD, EC 1.3.8.9	ACADVL	ACADV_HUMAN	25	5,394596647	0,000436364	Yes	1,65	Yes	1,55	Yes	1,56	Yes	1,74

Krebs Cycle enzymes							DOX_48h vs. Control		DOX_R 72h vs. Control		DOX_R 7d vs. Control		DOX_R 14d vs. Control	
**ID**	**Protein name**	**Gene Name**	**UniProt ID**	**Unique peptides**	**p-value**	**ANOVA q-value**	**Significant**	**Fold change**	**Significant**	**Fold change**	**Significant**	**Fold change**	**Significant**	**Fold change**
**P40926**	Malate dehydrogenase, mitochondrial, EC 1.1.1.37	MDH2	MDHM_HUMAN	14	4,78771387	0,000588235	Yes	1,21	Yes	1,47	Yes	1,40	Yes	1,30
**P36957**	Dihydrolipoyllysine-residue succinyltransferase component of 2-oxoglutarate dehydrogenase complex,	DLST	ODO2_HUMAN	8	1,22833636	0,044753664	No	1,41	No	1,18	No	1,56	No	1,31
**P48735**	Isocitrate dehydrogenase [NADP], mitochondrial, IDH, EC 1.1.1.42	IDH2	IDHP_HUMAN	23	5,999604474	0,0005	Yes	1,33	Yes	1,28	Yes	1,36	Yes	1,26
**O43837**	Isocitrate dehydrogenase [NAD] subunit beta, mitochondrial	IDH3B	IDH3B_HUMAN	14	3,508906962	0,001138824	Yes	1,32	Yes	1,55	Yes	1,23	No	1,22
**P51553**	Isocitrate dehydrogenase [NAD] subunit gamma, mitochondrial	IDH3G	IDH3G_HUMAN	10	2,193648846	0,006326711	No	1,02	No	1,25	Yes	1,59	No	1,45
**P50213**	Isocitrate dehydrogenase [NAD] subunit alpha, mitochondrial, EC 1.1.1.41	IDH3A	IDH3A_HUMAN	12	3,659003676	0,001068783	No	1,23	Yes	1,37	Yes	1,54	Yes	1,48
**Q99798**	Aconitate hydratase, mitochondrial, Aconitase, EC 4.2.1.3	ACO2	ACON_HUMAN	40	4,9188934	0,000525	Yes	1,50	Yes	1,72	Yes	1,95	Yes	2,06

OXPHOS involved proteins							DOX_48h vs. Control		DOX_R 72h vs. Control		DOX_R 7d vs. Control		DOX_R 14d vs. Control	
**ID**	**Protein name**	**Gene Name**	**UniProt ID**	**Unique peptides**	**p-value**	**ANOVA q-value**	**Significant**	**Fold change**	**Significant**	**Fold change**	**Significant**	**Fold change**	**Significant**	**Fold change**
**P28331**	NADH-ubiquinone oxidoreductase 75 kDa subunit, mitochondrial, EC 7.1.1.2	NDUFS1	NDUS1_HUMAN	20	1,711615705	0,016017406	No	-1,02	No	1,11	Yes	1,29	No	1,05
**P14927**	Cytochrome b-c1 complex subunit 7	UQCRB	QCR7_HUMAN	7	3,769681249	0,000991354	Yes	1,50	Yes	1,22	Yes	1,34	No	1,21
**P47985**	Cytochrome b-c1 complex subunit Rieske, mitochondrial, EC 7.1.1.8	UQCRFS1	UCRI_HUMAN	9	3,22941426	0,001533597	Yes	1,42	Yes	1,21	Yes	1,24	No	1,06
**P31930**	Cytochrome b-c1 complex subunit 1, mitochondrial	UQCRC1	QCR1_HUMAN	17	3,921081081	0,000923077	Yes	1,29	Yes	1,49	Yes	1,71	Yes	1,37
**P22695**	Cytochrome b-c1 complex subunit 2, mitochondrial	UQCRC2	QCR2_HUMAN	12	1,441508648	0,028496148	No	1,10	No	1,26	Yes	1,33	No	1,12
**O43169**	Cytochrome b5 type B	CYB5B	CYB5B_HUMAN	6	2,200078415	0,006245847	No	1,16	No	-1,05	No	1,39	Yes	1,35
**P31040**	Succinate dehydrogenase [ubiquinone] flavoprotein subunit, mitochondrial, EC 1.3.5	SDHA	SDHA_HUMAN	20	1,775875046	0,014121929	No	1,30	No	1,36	Yes	1,46	Yes	1,45
**Q96IX5**	ATP synthase membrane subunit K, mitochondrial	ATP5MK	ATPMK_HUMAN	3	1,278089971	0,0400199	No	1,00	No	1,11	No	1,11	No	1,14
**Q13423**	NAD(P) transhydrogenase, mitochondrial, EC 7.1.1.1	NNT	NNTM_HUMAN	38	4,123530594	0,000852941	No	1,12	No	1,16	Yes	1,53	Yes	1,62
**P38117**	Electron transfer flavoprotein subunit beta, Beta-ETF	ETFB	ETFB_HUMAN	15	5,49713171	0,000494845	Yes	1,24	Yes	1,34	Yes	1,34	Yes	1,55
**Q9NZ45**	CDGSH iron-sulfur domain-containing protein 1 (MitoNEET)	CISD1	CISD1_HUMAN	6	1,548583967	0,022599034	No	1,31	No	1,42	No	1,49	Yes	1,77

Redox homeostasis proteins							DOX_48h vs. Control		DOX_R 72h vs. Control		DOX_R 7d vs. Control		DOX_R 14d vs. Control	
**ID**	**Protein name**	**Gene Name**	**UniProt ID**	**Unique peptides**	**p-value**	**ANOVA q-value**	**Significant**	**Fold change**	**Significant**	**Fold change**	**Significant**	**Fold change**	**Significant**	**Fold change**
**P04040**	Catalase, EC 1.11.1.6	CAT	CATA_HUMAN	24	4,272142714	0,000760331	Yes	1,52	Yes	1,31	Yes	1,50	Yes	1,66
**P04179**	Superoxide dismutase [Mn], mitochondrial, EC 1.15.1.1	SOD2	SODM_HUMAN	12	9,556046184	0	Yes	2,02	Yes	2,18	Yes	3,02	Yes	3,54
**P00441**	Superoxide dismutase [Cu-Zn], EC 1.15.1.1	SOD1	SODC_HUMAN	7	1,486187248	0,025858934	Yes	1,33	No	1,13	No	1,01	No	1,13
**Q9Y2Q3**	Glutathione S-transferase kappa 1, EC 2.5.1.18	GSTK1	GSTK1_HUMAN	9	1,517965539	0,024146264	No	1,17	Yes	1,34	No	1,20	No	1,29
**P78417**	Glutathione S-transferase omega-1, GSTO-1, EC 2.5.1.18	GSTO1	GSTO1_HUMAN	21	1,720598725	0,015750439	No	1,02	No	1,08	Yes	1,19	No	1,19
**P30044**	Peroxiredoxin-5, mitochondrial, EC 1.11.1.24	PRDX5	PRDX5_HUMAN	14	3,735808802	0,001042493	Yes	1,47	Yes	1,41	Yes	1,30	Yes	1,30
**P35754**	Glutaredoxin-1 (Thioltransferase-1, TTase-1)	GLRX	GLRX1_HUMAN	4	2,938928718	0,002246305	Yes	1,63	Yes	1,47	No	1,24	No	1,24
**Q9NUJ1**	Palmitoyl-protein thioesterase ABHD10, mitochondrial, EC 3.1.2.22	ABHD10	ABHDA_HUMAN	12	2,597486759	0,003397333	Yes	1,44	Yes	1,40	Yes	1,30	Yes	1,45
**Q13162**	Peroxiredoxin-4, EC 1.11.1.24 (	PRDX4	PRDX4_HUMAN	13	3,543575097	0,001156863	No	1,15	No	1,15	Yes	1,30	Yes	1,35
**O95571**	Persulfide dioxygenase ETHE1, mitochondrial, EC 1.13.11.18	ETHE1	ETHE1_HUMAN	8	3,850533863	0,000969325	No	1,06	No	1,16	Yes	1,43	Yes	1,72
**Q96C36**	Pyrroline-5-carboxylate reductase 2, EC 1.5.1.2	PYCR2	P5CR2_HUMAN	9	3,935752773	0,000929032	Yes	1,62	Yes	1,65	No	1,22	Yes	1,75
**P32322**	Pyrroline-5-carboxylate reductase 1, mitochondrial, EC 1.5.1.2	PYCR1	P5CR1_HUMAN	11	2,778353565	0,002542222	No	1,17	Yes	1,24	No	1,17	Yes	1,30

Doxorubicin metabolizing enzymes							DOX_48h vs. Control		DOX_R 72h vs. Control		DOX_R 7d vs. Control		DOX_R 14d vs. Control	
**ID**	**Protein name**	**Gene Name**	**UniProt ID**	**Unique peptides**	**p-value**	**ANOVA q-value**	**Significant**	**Fold change**	**Significant**	**Fold change**	**Significant**	**Fold change**	**Significant**	**Fold change**
**P15121**	Aldo-keto reductase family 1 member B1, EC 1.1.1.300	AKR1B1	ALDR_HUMAN	22	8,128885746	0	Yes	1,65	Yes	1,65	Yes	2,13	Yes	2,13
**P14550**	Aldo-keto reductase family 1 member A1, EC 1.1.1.2	AKR1A1	AK1A1_HUMAN	21	5,862108398	0,000555556	Yes	1,17	Yes	1,24	Yes	1,34	Yes	1,50
**O75828**	Carbonyl reductase [NADPH] 3, EC 1.1.1.184	CBR3	CBR3_HUMAN	13	2,873215705	0,002346334	No	1,21	No	-1,03	No	1,16	Yes	1,48
**P16152**	Carbonyl reductase [NADPH] 1, EC 1.1.1.184	CBR1	CBR1_HUMAN	13	2,809274101	0,00245827	No	1,05	No	1,06	No	1,03	Yes	1,29

NAD(P)H homeostasis proteins							DOX_48h vs. Control		DOX_R 72h vs. Control		DOX_R 7d vs. Control		DOX_R 14d vs. Control	
**ID**	**Protein name**	**Gene Name**	**UniProt ID**	**Unique peptides**	**p-value**	**ANOVA q-value**	**Significant**	**Fold change**	**Significant**	**Fold change**	**Significant**	**Fold change**	**Significant**	**Fold change**
**Q13423**	NAD(P) transhydrogenase, mitochondrial, EC 7.1.1.1	NNT	NNTM_HUMAN	38	4,123530594	0,000852941	No	1,12	No	1,16	Yes	1,53	Yes	1,62
**P48735**	Isocitrate dehydrogenase [NADP], mitochondrial, IDH, EC 1.1.1.42	IDH1	IDHP_HUMAN	23	5,999604474	0,0005	Yes	1,33	Yes	1,28	Yes	1,36	Yes	1,26
**Q8NCW5**	NAD(P)H-hydrate epimerase, EC 5.1.99.6	NAXE	NNRE_HUMAN	12	2,886702261	0,002333333	No	1,23	Yes	1,35	Yes	1,51	Yes	1,50

Senescence involved proteins							DOX_48h vs. Control		DOX_R 72h vs. Control		DOX_R 7d vs. Control		DOX_R 14d vs. Control	
**ID**	**Protein name**	**Gene Name**	**UniProt ID**	**Unique peptides**	**p-value**	**ANOVA q-value**	**Significant**	**Fold change**	**Significant**	**Fold change**	**Significant**	**Fold change**	**Significant**	**Fold change**
**P16278**	Beta-galactosidase, EC 3.2.1.23	GLB1	BGAL_HUMAN	13	2,862544691	0,002354037	No	1,47	No	1,38	Yes	1,76	Yes	2,25

ATP/ADP homeostasis management							DOX_48h vs. Control		DOX_R 72h vs. Control		DOX_R 7d vs. Control		DOX_R 14d vs. Control	
**ID**	**Protein name**	**Gene Name**	**UniProt ID**	**Unique peptides**	**p-value**	**ANOVA q-value**	**Significant**	**Fold change**	**Significant**	**Fold change**	**Significant**	**Fold change**	**Significant**	**Fold change**
**Q9UIJ7**	GTP:AMP phosphotransferase AK3, mitochondrial, EC 2.7.4.10	AK3	KAD3_HUMAN	15	3,397917927	0,001306667	Yes	1,85	Yes	1,62	Yes	1,60	Yes	1,88
**P54819**	Adenylate kinase 2, mitochondrial, AK 2, EC 2.7.4.3	AK2	KAD2_HUMAN	11	2,1044092	0,007457052	Yes	1,34	Yes	1,26	No	1,20	No	1,03
**P00568**	Adenylate kinase isoenzyme 1, AK 1, EC 2.7.4.3	AK1	KAD1_HUMAN	7	1,387759523	0,031738542	Yes	1,37	No	1,19	No	1,24	No	1,12

Unsaturated fatty acids β-oxidation							DOX_48h vs. Control		DOX_R 72h vs. Control		DOX_R 7d vs. Control		DOX_R 14d vs. Control	
**ID**	**Protein name**	**Gene Name**	**UniProt ID**	**Unique peptides**	**p-value**	**ANOVA q-value**	**Significant**	**Fold change**	**Significant**	**Fold change**	**Significant**	**Fold change**	**Significant**	**Fold change**
**Q13011**	Delta(3,5)-Delta(2,4)-dienoyl-CoA isomerase, mitochondrial, EC 5.3.3.-	ECH1	ECH1_HUMAN	11	10,21329378	0	Yes	1,66	Yes	1,49	Yes	1,56	Yes	1,60
**O75521**	Enoyl-CoA delta isomerase 2, EC 5.3.3.8	ECI2	ECI2_HUMAN	11	1,824316086	0,01282397	Yes	1,37	No	1,21	Yes	1,41	No	1,31

Carnitine-supported fatty acid transport							DOX_48h vs. Control		DOX_R 72h vs. Control		DOX_R 7d vs. Control		DOX_R 14d vs. Control	
**ID**	**Protein name**	**Gene Name**	**UniProt ID**	**Unique peptides**	**p-value**	**ANOVA q-value**	**Significant**	**Fold change**	**Significant**	**Fold change**	**Significant**	**Fold change**	**Significant**	**Fold change**
**Q9UKG9**	Peroxisomal carnitine O-octanoyltransferase, COT, EC 2.3.1.137	CROT	OCTC_HUMAN	13	3,807935004	0,000967164	No	1,02	Yes	-1,19	Yes	1,27	Yes	1,26
**O43772**	Mitochondrial carnitine/acylcarnitine carrier protein SLC25A20	SLC25A20	MCAT_HUMAN	7	1,64250647	0,018286195	No	1,32	No	1,61	No	2,11	No	2,12
**P23786**	Carnitine O-palmitoyltransferase 2, mitochondrial, EC 2.3.1.21	CPT2	CPT2_HUMAN	14	1,441183745	0,028492687	No	2,72	No	2,78	Yes	3,53	No	2,95
**P50416**	Carnitine O-palmitoyltransferase 1, liver isoform, CPT1-L, EC 2.3.1.21	CPT1A	CPT1A_HUMAN	23	5,829134293	0,000586667	No	1,27	Yes	1,46	Yes	1,94	Yes	2,31
